# Head to head randomized trial of two decision aids for prostate cancer

**DOI:** 10.1186/s12911-021-01505-x

**Published:** 2021-05-12

**Authors:** Angela Fagerlin, Margaret Holmes-Rovner, Timothy P. Hofer, David Rovner, Stewart C. Alexander, Sara J. Knight, Bruce S. Ling, James A.Tulsky, John T. Wei, Khaled Hafez, Valerie C. Kahn, Daniel Connochie, Jeffery Gingrich, Peter A. Ubel

**Affiliations:** 1 Salt Lake City VA Informatics Decision-Enhancement and Analytic Sciences (IDEAS) Center for Innovation, Salt Lake City, UT USA; 2 Department of Population Health Sciences, University of Utah, 295 Chipeta Way Rm 1S105, Salt Lake City, UT 84132 USA; 3 Center for Ethics and Department of Medicine, Michigan State University, East Lansing, MI USA; 4 Ann Arbor VA HSR&D Center for Practice Management and Outcomes Research, Ann Arbor, MI USA; 5 Department of Internal Medicine, University of Michigan, Ann Arbor, MI USA; 6 Department of Consumer Science, Purdue University, West Lafayette, IN USA; 7 Department of Internal Medicine, University of Utah, Salt Lake City, UT USA; 8 Department of Internal Medicine, University of Pittsburgh, Pittsburgh, PA USA; 9 Department of Psychosocial Oncology and Palliative Care, Dana-Farber Cancer Institute, Boston, MA USA; 10 Division of Palliative Medicine, Department of Medicine, Brigham and Women’s Hospital, Boston, MA USA; 11 Department of Urology, University of Michigan, Ann Arbor, MI USA; 12 Center for Bioethics and Social Sciences in Medicine, University of Michigan, Ann Arbor, USA; 13 Division of Urology, Department of Surgery, Duke University, Durham, NC USA; 14 Sanford School of Public Policy, Duke University, Durham, NC USA; 15 Fuqua School of Business, Duke University, Durham, USA

**Keywords:** Decision aids, Prostate cancer, Patient education, Literacy, Plain language, Shared decision making

## Abstract

**Background:**

While many studies have tested the impact of a decision aid (DA) compared to not receiving any DA, far fewer have tested how different types of DAs affect key outcomes such as treatment choice, patient–provider communication, or decision process/satisfaction. This study tested the impact of a complex medical oriented DA compared to a more simplistic decision aid designed to encourage shared decision making in men with clinically localized prostate cancer.

**Methods:**

1028 men at 4 VA hospitals were recruited after a scheduled prostate biopsy. Participants completed baseline measures and were randomized to receive either a simple or complex DA. Participants were men with clinically localized cancer (N = 285) by biopsy and who completed a baseline survey. Survey measures: baseline (biopsy); immediately prior to seeing the physician for biopsy results (pre- encounter); one week following the physician visit (post-encounter). Outcome measures included treatment preference and treatment received, knowledge, preference for shared decision making, decision making process, and patients’ use and satisfaction with the DA.

**Results:**

Participants who received the simple DA had greater interest in shared decision making after reading the DA (p = 0.03), found the DA more helpful (p’s < 0.01) and were more likely to be considering watchful waiting (p = 0.03) compared to those receiving the complex DA at Time 2. While these differences were present before patients saw their urologists, there was no difference between groups in the treatment patients received.

**Conclusions:**

The simple DA led to increased desire for shared decision making and for less aggressive treatment. However, these differences disappeared following the physician visit, which appeared to change patients’ treatment preferences.

*Trial registration* This trial was pre-registered prior to recruitment of participants.

**Supplementary Information:**

The online version contains supplementary material available at 10.1186/s12911-021-01505-x.

## Background

Patient decision aids (DAs) designed to help patients diagnosed with prostate cancer become more informed and involved with their prostate cancer treatment were first designed and evaluated in 1988 [1, 2]. DAs are typically focused on diagnoses that have clinical equipoise, meaning that the treatment options are equivalent in terms of survival, but have different side effects associated with treatment. Prostate cancer is an excellent example of clinical equipoise in that life expectancy is almost equivalent across treatment options (active surveillance, radiation therapy, and prostatectomy) [3, 4], but the risks and types of side effects are different (e.g., bladder and bowel dysfunction for those who receive radiation or prostatectomy, while surveillance requires frequent follow-up testing and may cause anxiety about living with cancer [5–8]). A recent decision analysis of patients with clinically localized prostate cancer concluded that for 65-year- old men in average health, surgery resulted in 0.3 additional years of life expectancy at the expense of 1.6 additional years of impotence or incontinence, a net difference of 0.05 fewer quality adjusted life-years.

Systematic reviews of DAs show they increase patient knowledge, increase patient clarity about their own values, decrease decisional conflict, and increase patient interest in active roles in decision making [1]. However, the focus and quality of these tools and their impact on treatment preferences and treatment received is highly variable [1, 9]. Explanations for this variability are not well understood. Potential sources of variability identified previously include differences in DA content, in DA use as preparation for the encounter vs. during the encounter vs. following the encounter, and characteristics of populations such as numeracy, literacy and education [1]. With growing support for shared decision making in practice guidelines and continued development of new DAs, it is important to understand, in real world settings, how DAs vary in their impact on treatment process and patient preference as well as long-term impact on treatment received.

There have been a number of studies that have evaluated the use of a decision aid for patients diagnosed with prostate cancer. In a 2015 meta-analysis, 13 studies with prostate cancer patients were analyzed [10]. In most of these studies, the authors compared key outcomes between those who received a decision aid and those who received usual care (i.e., no supplemental materials provide to the patient) generic information. However, two studies did compare two types of decision aids. Our study follows in the tradition of these two studies, in that we compare two decision aids that differed in design features.

Findings across studies testing prostate cancer decision aids with patients have varied, but generally the overall pattern of results are similar to previous work described above in regards to decision aids in general [10]. Specifically, patients who received a decision aid were slightly more knowledgeable [11, 12], more satisfied with their decision [11, 13], and had lower decisional conflict (though several studies show no impact on decisional conflict, results were inconsistent) [11, 13–15]. The two studies that compared decision aids (as we do in the current study) did not show any differences in decisional conflict [16, 17]. Similarly, there were inconsistent and small effect sizes when comparing preparation for decision making [16–21] and satisfaction with patient–physician communication [12, 22]. None of the tools were shown to have an impact on treatment decisions (either deferring treatment vs. immediate treatment nor type of intervention) [13, 14, 18, 23–25]. More recent findings have found little impact of prostate cancer decision aids; with no impact of a tool in improving knowledge, involvement in decision making, or decisional conflict [26].

Additionally, they found that overall information satisfaction was lower in those receiving the decision aid. However, van Tol-Geerdink et al., found a decision aid impacted treatment preferences in those receiving a decision aid (i.e., increase in brachytherapy and decrease in Dutch patients who were undecided) [24].

In this trial we compared two existing DAs introduced into routine urology practice [9, 27, 28]. The goal of the study was to determine the impact of a simple decision aid compared to a complex DA on treatment preference and decision processes. The tools differed in their use of plain language, their encouragement of shared decision making, and their use of patient experiences (in the form of testimonials; see Table 1).

**Table 1:** Differences between decision aids

Simple DA	Complex DA
Comparison of benefits and side effects of treatment	Comparison of benefits and side effects of treatment
Encouragement of active role in shared decision making	Little discussion of shared decision making
Plain language (7th grade reading level)	Standard language (> 9th grade reading level)
Patient testimonials	None
Risks and benefits described as number of people out of 100	Side effect rates described as percentages
IPDASi score: 66/100	IPDASi score: 31/100

The simple DA was developed by the Michigan Cancer Consortium (MCC), led in part by Drs. Fagerlin, Holmes-Rovner, Rovner, and Wei [27, 29]. The development of the tool included a needs assessment conducted via a literature review of available decision aids [9] and through long discussions with members of the Michigan Cancer Consortium’s Prostate Cancer Action Committee (which included patients, urologists, radiation oncologists, medical oncologists, and others). With two plain language experts, we designed the tool and received feedback from former prostate cancer and BPH patients (naïve to prostate cancer, so they could reflect how people with little prior knowledge of prostate cancer would react to the tool) [29]. Primary care physicians, radiation oncologists, urologists, and urological nurses reviewed the tool and provided feedback to ensure accuracy and balance across the different treatments. The comparison DA by the National Comprehensive Cancer Network (NCCN) and the American Cancer Society [28] was chosen because of our previous work showing it was one of the best available tools [9] and its professional credibility.

We studied the decision-making process in men with clinically localized prostate cancer at four geographically dispersed Veterans Affairs clinical sites. The objective of this study is to determine how decision aids with different components, including literacy level, use of testimonials, encouragement of shared decision making, and strategies for patient–provider communication, differently impact patients’ knowledge, the treatments that patients receive, their experience with the decision making process, and their satisfaction with the tool. Specifically, we asked the following primary research questions: Would the two tools differ on impact on: (1) shared decision making, (2) knowledge, (3) satisfaction with the decision aid and (4) impact on the decision process and treatment received.

## Methods

### Objectives and participants

This randomized trial was designed to contribute understanding of the variability of impact in routine practice of DAs on patient treatment preference attributable to differences in DA content and readability. We chose two DAs for a head-to-head trial (1:1 allocation ratio) of a “simple” DA and a “complex” DA. The purpose was to investigate the impact of two previously developed and publicly available decision aids [27, 28]. To capture the process of treatment decision making, patients participated from biopsy through the diagnostic clinical encounter. Patients were enrolled by research assistants sequentially after being scheduled for a prostate biopsy. Research assistants also assigned participation to study arm.

Participants were deemed eligible if they had a prostate biopsy scheduled. Research staff approached patients at the time of biopsy and invited them to participate in a study to evaluate the DAs. Those who provided informed consent were included in the study and were randomized to receive one of the DAs. The analytic sample was all patients in the study whose biopsy showed clinically localized prostate cancer (Gleason score 6 or 7, PSA < 20 ng/ml). Patients who had no evidence of cancer or more advanced cancer were not included beyond the initial biopsy visit and their data is not included in this report. Physician participants were urology residents and attending physicians whose patients participated in the study. Treating physicians did not receive any training in shared decision making or in the use of DAs. They were told that patients had received a DA booklet, but were not asked to alter their practice in any way.

Physicians provided demographic data at their recruitment. The trial was conducted at four Veterans Administration (VA) Centers geographically distributed across the United States (Ann Arbor, Durham, Pittsburgh, and San Francisco). VA clinics are publicly supported facilities serving people who had performed military service. Recruitment began in September 2008 in Ann Arbor and in the fall of 2009 in Durham, Pittsburgh, and San Francisco. Recruitment concluded in May of 2012 at all sites.

They serve a broad population, with an over-representation of patients of moderate to low income, since the VA provides care without regard to ability to pay. The study was approved by the VA Institutional Review Board (IRB) at each participating site; written informed consent was obtained from each patient and physician participant. The funding agencies had no role in conduct or reporting of the study. Each site’s local IRB approved the study and written informed consent was obtained from participants. The study adheres to CONSORT reporting guidelines.

### Intervention

Following the baseline survey (biopsy survey/time 1), patients in the analytic sample completed surveys immediately before the physician encounter (pre-encounter survey/time 2) and approximately 7–10 days following the physician encounter (post-encounter survey/time 3). Surveys were read aloud by research staff. Research staff telephoned patients two days before the physician encounter to remind them to read the DA, but did not inform patients of the diagnosis. Patients learned the diagnosis from their physician, with the exception of one site that followed a practice of giving the diagnosis over the telephone. Participants at that site were surveyed before the diagnosis phone call. Patients were also asked to participate in audio recording of the physician encounter at which biopsy results and initial treatment options were discussed. (Qualitative data results have been reported previously [30–32]). PSA levels, Gleason Scores, and treatment received were obtained from electronic medical records.

### Decision aids

Both DAs were previously developed for use in an unselected population of men with prostate cancer. Both were publicly available at no charge. The simple DA was developed by the Michigan Cancer Consortium (MCC) [27, 29]. The comparator DA, developed by the National Comprehensive Cancer Network and the American Cancer Society [28], was chosen because of its high quality information [9] and credibility. Both DAs aimed to guide treatment decisions, comparing the likelihood of benefits and side effects of treatments. Both decision aids used the terminology “watchful waiting” because active surveillance was not a commonly used term when this study began. During the time the study was conducted, watchful waiting terminology was replaced by active surveillance. Watchful Waiting, as we use it, means to do no therapy until symptoms occur. Active Surveillance includes periodic PSA tests, biopsies, and other diagnostic maneuvers. The MCC DA used plain language and reflected many of the standards of the International Patient Decision Aids Consortium (IPDAS), although the tool was developed prior to the publication of the IPDAS standards [29, 33–35]. The NCCN standard language DA was chosen because of its high-quality information about risks and benefits of alternative treatments for prostate cancer, the decision guidance and the high credibility of the sponsoring organizations. Both DAs are provided as additional files.

The simple DA, “Making the Choice: Deciding What to Do About Early Stage Prostate Cancer” incorporated text and document layout features to support comprehension [34, 35]. The use of plain language was based on the extensive literature showing that plain language materials can improve patient understanding and adopt necessary services (e.g., vaccines) [36–40].

Additionally, “Making the choice” used patient testimonials to convey the message that each man should make the decision that was right for him. The DA described the number of people out of 100 who are likely to experience specific risks and benefits presented in a table. The NCCN DA “Prostate Cancer: Treatment Guidelines for Patients” was designed to provide treatment guidelines for patients with either early or later stage prostate cancer. The information in the DA was based on the NCCN’s Clinical Practice Guidelines. It included common side effect rates in percentages, and used decision trees to present treatment options.

#### A priori* comparison of DAs*

Inclusion of content topics of the two decision aids was similar. Key differences were inclusion of patient testimonials and information about the patient experience in the simple DA, and detail about treatment of advanced cancer in the complex DA. The two DAs were independently scored for literacy using the Suitability Assessment of Materials [9, 41] and were compared using IPDASi, a DA quality scoring system that evaluates the quality of the DAs across 9 dimensions (development, disclosure of potential conflicts of interest, evaluation of the instrument, evidence, decision guidance, information about pros and cons of alternatives, including no action, plain language, inclusion of probabilities of outcomes and values) [42]. The complex DA was scored as having greater than a 9th grade reading level, whereas the simple tool was evaluated at a 7th grade reading level. A summative assessment of the quality of the two DAs was obtained by having independent raters use the IPDASi scoring system, which applies the IPDAS quality criteria [33, 42]. Items within the 9 domains were scored on a 4-point scale (1 = lowest and 4 = highest). The mean adjusted score for each domain is presented as a value out of 100. The simple DA scored 66/100; the standard language DA scored 31/100 on the IPDASi evaluation. The complex DA score, in part, reflects lack of data about background and pilot testing. The substantive differences were in the dimensions of information about pros and cons of each alternative, plain language and decision guidance, which favored the simple DA.

### Outcomes

Our primary outcome was focused on the treatment patients received (via data collected from electronic health record). We chose this as our primary outcome based on the Ottawa Decision Framework [43] which asserts that decision support tools improve the quality of the decision (based on knowledge and values) as well as the impact on the decision and the implementation of the decision. We also measured patients’ treatment preference prior to their diagnosis (pre-encounter) and following their diagnosis (post-encounter). Treatment preference at the preclinical encounter refers to the treatment the patient preferred, rather than preference for outcomes, using the following question: “Although you may not have cancer, we would like to know what treatment you think you might have if you were to have prostate cancer.” Participants were read a list of treatments (surgery, external beam radiation, brachytherapy, watchful waiting, adjuvant hormone therapy, experimental therapies) and participants answered yes or no to each. Preferences for multiple treatments were allowed since patients had not seen the physician nor received a diagnosis at the pre-encounter survey. While we measured patient preferences following their physician visit, we do not report them here as we have chosen to only report the treatment they actually received (at the post-encounter survey, some were still unsure of choice and there were no differences in pattern of results between the post-encounter survey and treatment received).

Decision process outcomes included early-stage prostate cancer treatment knowledge, interest in shared decision making, perception of patient–physician communication, use/satisfaction with DA and prostate cancer specific anxiety. The *prostate cancer treatment knowledge scale* was administered at the pre and post encounter surveys and was composed of seven questions derived from a survey of newly diagnosed prostate cancer patients [29, 44] and questions adapted from Lee et al. [45, 46] Questions addressed the survival benefit and side effects associated with treatments. *Interest in shared decision making* was measured at all three time points and used a prostate cancer adaptation of Degner and Sloan's [47] Control Preference Scale which asks people whether they: (1) prefer to make the final treatment decision; (2) prefer to make the final selection of their treatment after considering their doctor’s opinion; (3) prefer that their doctor share responsibility; (4) prefer that their doctor make the final decision after considering their opinion; or (5) prefer to leave all treatment decisions to their doctor.

*Perception of patient–physician* communication was measured using two scales and were administered at the post-encounter survey. COMRADE (Combined Outcome Measure for Risk communication And treatment Decision making Effectiveness) is a 20-item patient-based outcome measure for evaluation of treatment decision making and satisfaction with communication, validated for use in clinical encounters [48]. It contains 2 sub-scales: 1) satisfaction with physician communication and 2) patient confidence in the decision that was made. The scale also has good internal consistency (Cronbach’s alpha = 0.92). Perception of patient–physician communication was measured using Lerman et al.’s patients’ Perceived Involvement Care Scale (PICS) [49], which measures the level of information exchange between the physician and themselves and their participation in decision making.

Additionally, we asked several questions about whether the urologist provided a recommendation, what the recommendation was, how strong it was, and how influential it was (measured at post-encounter interview). Use of and satisfaction with the decision aid was an investigator developed set of seven questions which assessed participants’ perception and use of the DA (e.g., time spent reading DA, influence and helpfulness of DA; see Table 2 for questions) and were administered at the pre-encounter survey. Anxiety was measured at each time point using a subset of the Memorial Anxiety Scale for Prostate Cancer (MAX-PC) [50]. This 18-item scale has a high degree of internal consistency (α = 0.89), test–retest reliability (α = 0.89) and concurrent validity (r’s between 0.45-0.57 on subscales). Prostate cancer specific anxiety was included as an outcome measure because a persistent concern in the field is that receiving detailed disease and treatment specific information might increase patient anxiety. Process variables were chosen because all are aspects of decision-making that should affect reaching an informed and shared decision [1].

**Table 2:** Differences in process outcomes by decision aid received

	N	Complex DA	Simple DA	Abs. diff/(CI)	t/(p value)
Knowledge (proportion correct,12 item scale)	279	0.48	0.53	0.05 (− 0.01, 0.11)	1.57 (0.116)
Interest in shared decision making					
Biopsy interview	1012	3.30	3.34	0.04 (− 0.05, 0.14)	0.88 (0.380)
Pre-clinic interview	281	3.33	3.50	0.16* (0.02, 0.31)	2.20 (0.027)
Post-clinic interview	241	3.50	3.60	0.10 (− 0.06, 0.26)	1.19 (0.235)
Anxiety					
Biopsy interview	1005	1.02	1.07	0.05 (− 0.04, 0.14)	1.06 (0.290)
Pre-clinic interview	280	1.08	1.12	0.03 (− 0.13, 0.20)	0.39 (0.694)
Post-clinic interview	240	2.08	2.15	0.07 (− 0.12, 0.25)	0.72 (0.474)
How helpful was the DA					
Influencing treatment	272	3.02	3.32	0.31 (− 0.02, 0.63)	1.86 (0.063)
Understanding prostate cancer	272	3.76	4.13	0.38** (0.11, 0.64)	2.78 (0.005)
Understanding treatment options	272	3.49	3.89	0.40** (0.14, 0.67)	2.96 (0.003)
How much they liked the DA	272	3.77	4.09	0.32* (0.04, 0.59)	2.28 (0.022)
Patient–physician communication:					
COMRADE		2414.25	4.28	0.03 (− 0.13, 0.18)	0.31 (0.757)
PICS: MD facilitation		2391.69	1.75	0.06 (− 0.02, 0.13)	1.47 (0.141)

Results analyzed accounting for stratified randomization by site, race and literacy as random effects

^*^p < 0.05; **p < 0.01; ***p < .001

Demographic data were collected at the biopsy visit to allow testing of differences between intervention groups that might affect outcomes and bias the results. Patients’ race, ethnicity, age, marital status and education were collected. Literacy and numeracy were collected to test for differences between groups and to test outcomes across levels of literacy and numeracy**.** The literacy measure was the Rapid Estimate of Adult Literacy in Medicine (REALM) medical word recognition task, which produces a grade level score with ≤ 8 representing low literacy [51]. Numeracy was measured using the 8-item Subjective Numeracy Scale, which measures participants’ perceptions of mathematical skills [52, 53]. Low numeracy was defined as a score of 4.75 and below (approximate median split).

### Randomization and power

Patients receiving a prostate biopsy to test for cancer were randomized to receive one of two DAs that varied by shared decision-making intensity and literacy (simple vs. complex). A biostatistician assigned participants to study arms using block randomization in blocks of 2 and 4 stratified by race, literacy and site to ensure balance of African Americans and low literacy patients in each arm.

Randomization charts were developed for each site by the biostatistician. Although three strata for race (White, African American and Other Race) were used originally, due to very small numbers of subjects of other races randomized (with none at two sites) this stratum was collapsed into the White stratum for analysis. Allocation of patients to study arm was not revealed to providers, although since DAs were in booklet format, patients could bring DAs to the physician encounter. Neither physicians nor patients were told the hypothesis of the study.

Participants and research teams were blinded to outcome assessment. Power analyses showed a required 103 subjects per arm, using a hierarchical mixed-effect model and assuming a two-sided 0.05 level test with 80% power, a within-provider correlation of 0.10, and 10 patients per provider. Minimum important difference has not been established.

### Statistical analyses

Descriptive statistics (frequencies, means, standard deviations) were calculated for all outcome variables and patient demographics by intervention group. Given the stratified block randomization, all analyses were done accounting for the design [54]. For continuous, dichotomous, ordinal and polytomous outcomes, the results by group and the marginal treatment effect were estimated conditional on strata analyzed as random effects [55–57]. Test statistics and confidence intervals were calculated using the delta method [58]. Although using random effects seems preferable [57], the results were trivially changed when estimating the results accounting for the design using fixed effects, although three strata had to be dropped due to lack of variation in outcomes. In an alternative analysis, we analyzed the treatment received accounting for the MD seen as a random effect, although in this analysis it was not possible to also account for the design variables due to small cell sizes. Again, the results were only trivially different (i.e., the pattern of results were consistent). All analyses were performed using Stata 13.1 [59] and with the original assigned groups.

## Results

The mean age of the patient sample was 63.3 years (SD = 5.9); 33% were nonwhite, and 40% had high school education or less. The mean age of 45 treating physicians was 33 years (SD = 7.2);20% were female, and 34% were nonwhite. On average, each physician was audio recorded in 6 clinical encounters (SD = 4.3) and was 10 years post-graduation.

**Fig. 1:**
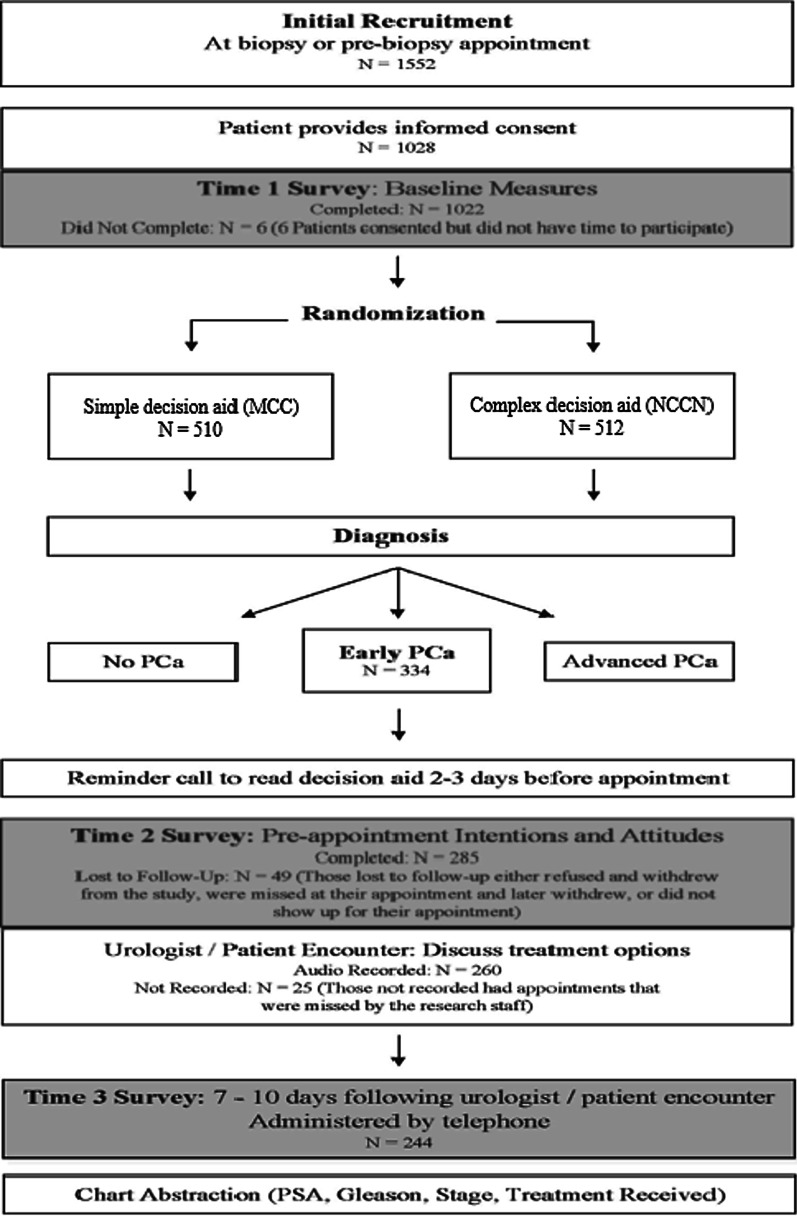
Study design

Figure 1 shows patients’ progression through the study. 1552 men who received a prostate biopsy to test for cancer were asked to participate in this study and 1028 agreed (66%). Of those, 1022 completed the biopsy survey (99%). Only 334 of those men were diagnosed with clinically localized prostate cancer (33%) and 285 of those completed the pre-encounter interview (85%), 244 completed the post-encounter interview (73%), and we were able to determine the treatment the patient received in 216 cases (65%). There was an equal distribution of subjects in each study arm across time points. At each time point, 50% of participants received each decision aid (simple DA N: T1 = 510, T2 = 141, T3 = 122; complex DA N: T1 = 512, T2 = 144, T3 = 122). Table 3 shows demographics across time points and study arm. There were no demographic or clinical differences among participants across study arms at any of the 3 time points. Recruitment occurred between September 2008 and May 2012, and ended when the enrollment goal was satisfied.

**Table 3:** Demographic characteristics of the sample

	Biopsy survey		Pre-encounter survey		Post-encounter survey	
	N (%)		N (%)		N (%)	
	Simple decision aid	Complex decision aid	Simple decision aid	Complex decision aid	Simple decision aid	Complex decision aid
	N = 510	N = 512	N = 141	N = 144	N = 122	N = 122
Age						
M (SD)	63.41 (5.86)	63.14 (6.02)	63.35 (5.99)	63.13 (6.22)	64.01 (5.78)	63.02 (6.06)
Race and ethnicity						
Caucasian	385 (75.6)	397 (77.5)	99 (70.2)	105 (72.9)	91 (74.6)	89 (73.0)
African American	115 (22.6)	106 (20.7)	39 (27.7)	36 (25.0)	29 (23.8)	29 (23.8)
American Indian or Alaskan Native	15 (2.9)	14 (2.7)	5 (3.5)	3 (2.1)	3 (2.5)	2 (1.6)
Pacific Islander or Native Hawaiian`	0 (0.0)	1 (0.2)	0 (0.0)	1 (0.7)	0 (0.0)	1 (0.8)
Asian	1 (0.2)	6 (1.2)	0 (0.0)	2 (1.4)	0 (0.0)	2 (1.6)
Hispanic	11 (2.2)	11 (2.1)	2 (1.4)	3 (2.1)	2 (1.6)	3 (2.5)
Middle Eastern	2 (0.4)	2 (0.4)	0 (0.0)	1 (0.7)	0 (0.0)	1 (0.8)
Education						
< High school	27 (5.3)	23 (4.5)	3 (2.1)	3 (2.1)	3 (2.5)	1 (0.8)
High school/GED	144 (28.4)	161 (31.5)	40 (28.4)	41 (28.5)	37 (30.3)	32 (26.2)
Trade school	21 (4.1)	25 (4.9)	4 (2.8)	8 (5.6)	4 (3.3)	7 (5.7)
Some college/Assoc	223 (44)	210 (41.1)	66 (46.9)	63 (43.8)	56 (45.9)	57 (46.7)
College degree	92 (18.1)	92 (18)	28 (19.9)	29 (20.2)	22 (18)	25 (20.5)
Marital status						
Married/partner	280 (55.2)	285 (55.9)	72 (51.4)	83 (57.6)	60 (49.6)	70 (57.4)
Divorced/separated	162 (32)	166 (32.6)	51 (36.5)	49 (34)	46 (38)	39 (31.9)
Widowed	27 (5.3)	18 (3.5)	4 (2.9)	4 (2.8)	3 (2.5)	4 (3.3)
Never married	38 (7.5)	41 (8.0)	13 (9.3)	8 (5.6)	12 (9.9)	9 (7.4)
Literacy						
Inadequate	140 (27.7)	139 (27.4)	34 (24.3)	41 (28.9)	30 (24.8)	32 (26.7)
Adequate	366 (72.3)	368 (72.6)	106 (75.7)	101 (71.1)	91 (75.2)	88 (73.3)
Numeracy						
M (SD)	4.57 (1.03)	4.56 (1.01)	4.69 (0.96)	4.69 (0.85)	4.64 (0.97)	4.75 (0.83)
Gleason score						
Gleason 6	72 (14.1)	72 (14.1)	72 (51.1)	72 (50.0)	59 (50.9)	54 (47.0)
Gleason 7	69 (13.5)	72 (14.1)	69 (48.9)	72 (50.0)	57 (49.1)	61 (53.0)
PSA						
M (SD)	5.98 (2.98)	6.12 (2.61)	5.98 (2.98)	6.12 (2.61)	6.25 (3.13)	6.07 (2.68)

Demographics for biopsy interview are all patients recruited into the study (text only includes patients ultimately diagnosed with cancer). PSA and Gleason scores were only extracted from electronic medical records for patients diagnosed with localized cancer

### Treatment preference

**Fig. 2:**
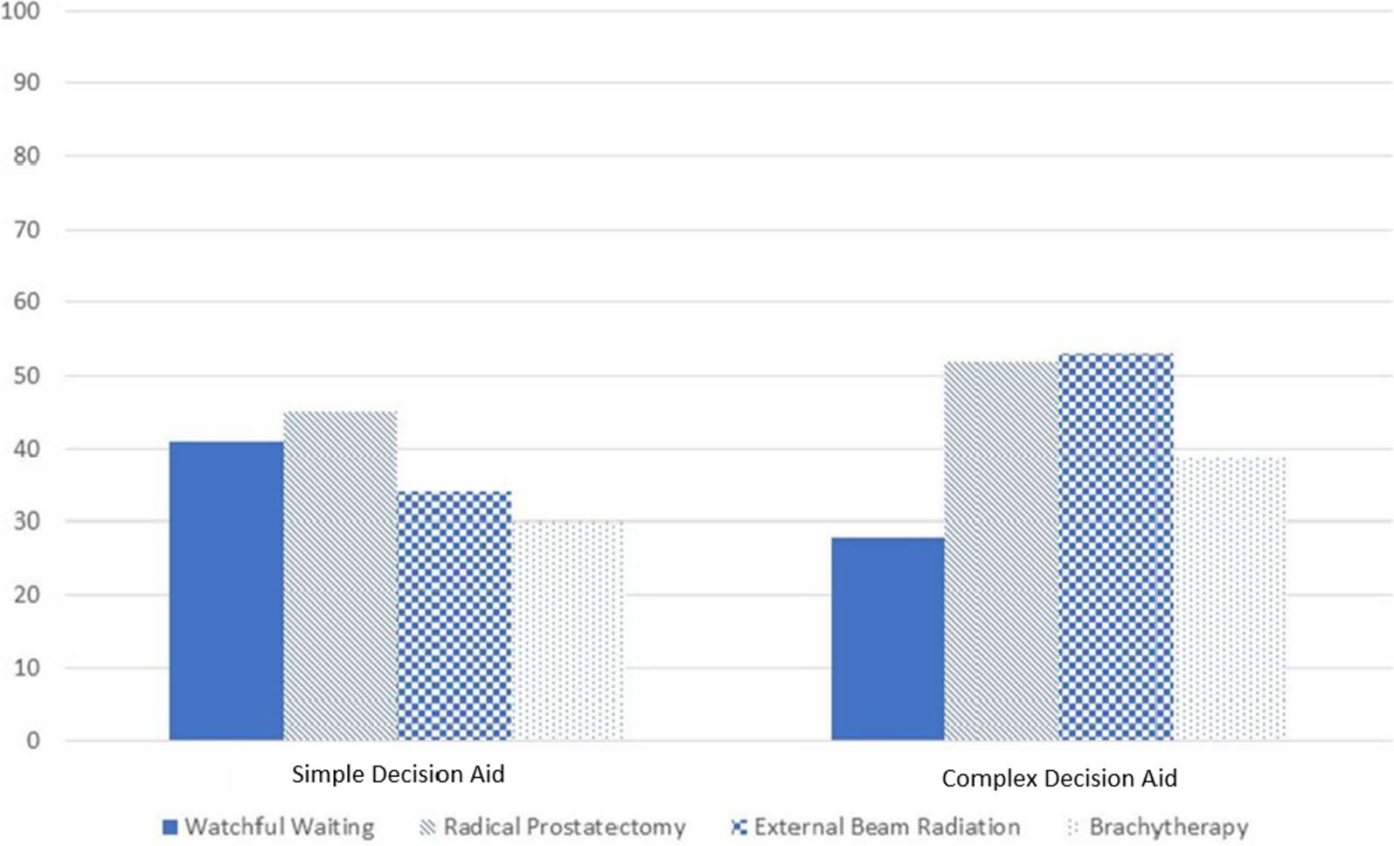
Percent people endorsing considering a treatment

As shown in Fig. 2, patients’ treatment preferences for external beam radiation and watchful waiting/active surveillance were different by study arm *prior* to meeting with the physician (i.e., Pre-encounter survey: z = -2.82, p = 0.005 and z = 2.22, p = 0.03 respectively). There were no differences in surgical or brachytherapy preferences (z = −1.06, p = 0.29 and z = -1.35, p = 0.18 respectively).

### Treatment received

Six months following diagnosis, the treatment that the patient received was extracted from the electronic medical record. The proportion who received surgery, radiation, and watchful waiting did not differ between the control and intervention groups (see Table 4).

**Table 4:** Treatment received by decision aid received (proportion in each category)

	Simple DA	Complex DA	Abs. Diff/(CI)	z/(p value)
Surgery	0.34	0.27	− 0.07 (− 0.19, 0.05)	− 1.17 (0.243)
Radiation	0.20	0.21	0.01 (− 0.09, 0.11)	0.22 (0.824)
Watchful waiting/active surveillance	0.46	0.52	0.06 (− 0.07, 0.19)	0.88 (0.380)

Results analyzed accounting for stratified randomization by site, race and literacy as random effects

^*^ *p* < 0.05; ** *p* < 0.01; *** p < .001

### Knowledge

There was no difference in prostate cancer specific knowledge between groups.

### Interest in shared decision making

There was no difference by DA in preference for making decisions at time of biopsy, (patient-centered: 3.34 vs. standard: 3.30, t = 0.88, p = 0.38; Table 2). However, at the pre-encounter interview, those receiving the simple DA were more interested in having an active role in the decision than those who received the complex DA (3.50 vs. 3.33, t = 2.20, p = 0.03). At the post-encounter interview, there was no difference in preference for decision participation (simple DA = 3.60 vs. complex DA = 3.50, t = 1.19, p = 0.24).

### Anxiety

Anxiety was low across patients in both arms. There was no difference by study arm at the biopsy, pre-encounter, or post-encounter surveys (all p’s > 0.20; see Table 2).

### Use of and satisfaction with decision aid

Those receiving the simple DA reported spending significantly less time reading the tool (see Table 5) and were more likely to have shared the decision aid with a partner (0.46 vs. 0.30, z = 2.87 p = 0.004; see Table 6). However, there were no differences between study arms in terms of whether they read the decision aid or brought it to the clinic (p’s > 0.15).

**Table 5:** Time spent looking at the decision aid (proportion in each category)

	Simple DA	Complex DA	Abs. diff/(CI)	z/(p value)
Less than 30 min	0.15	0.27	0.13*** (0.05, 0.20)	3.41 (0.001)
30–60 min	0.47	0.51	0.03 (− 0.00, 0.07)	1.76 (0.078)
1–2 h	0.25	0.15	− 0.09** (− 0.15, − 0.04)	− 3.22 (0.001)
More than 2 h	0.13	0.07	− 0.07** (− 0.11, − 0.02)	− 3.07 (0.002)

Results analyzed accounting for stratified randomization by site, race and literacy as random effects

^*^p < 0.05; **p < 0.01; ***p < .001

**Table 6:** Differences in process outcomes by decision aid received (proportion answering yes)

	N	Simple DA	Complex DA	Abs. diff/(CI)	z/(p value)
Did you read the DA	282	0.88	0.93	0.05 (− 0.02, 0.12)	1.39 (0.164)
Share DA					
Partner	273	0.29	0.46	0.17** (0.05, 0.28)	2.87 (0.004)
Other family	274	0.15	0.17	0.01 (− 0.07, 0.10)	0.33 (0.742)
Friend	274	0.14	0.15	0.02 (− 0.07, 0.10)	0.37 (0.713)
Bring DA to clinic	281	0.55	0.55	0.00 (− 0.13, 0.13)	0.05 (0.962)

Results analyzed accounting for stratified randomization by site, race and literacy as random effects

^*^p < 0.05; **p < 0.01; ***p < .001

The simple DA was reported to be more helpful in understanding prostate cancer (M’s = 4.13 vs. 3.76, t = 2.78, p = 0.005) and treatment options (M’s = 3.89 vs. 3.49, t = 3.05, p = 0.005). Participants receiving the simple DA were not more likely to say that the DA influenced their treatment preference (M’s = 3.32 vs. 3.02, t = 1.86, p = 0.06). Finally, those who received the simple DA reported liking it more (M’s = 4.09 vs. 3.77, t = 2.28, p = 0.02). There were no significant differences in satisfaction with the DA by literacy level across both arms (all p’s > 0.05).

### Satisfaction with physician communication

There were no differences by DA received in patients’ perceptions of communication with their urologist as measured by COMRADE and PICS during the post-survey encounter (see Table 2). We found both groups’ evaluation of the urologists’ communication to be good.

## Discussion

Our results show that the two DAs both functioned well to inform patients of their options, including details of treatments and their outcomes. There were no differences between study arms in whether they read the decision aid or brought it to the clinical encounter. As hypothesized, decision-making process variables show that the simple DA was more accessible and easier to understand. It was associated with higher interest in shared decision making (pre-encounter) and ease of DA use. Those receiving the simple DA reported spending significantly less time reading the tool to obtain the same knowledge, and were more likely to have shared the decision aid with a partner. The simple DA was also reported to be more helpful in understanding prostate cancer and treatment options and it received higher likability scores. Demographic and literacy variables that might have confounded the results were not different at the pre-encounter survey. We believe this is the first head-to-head comparison of DAs varying by literacy and SDM emphasis. While plain language writing has been recommended, our results suggest it may impact patients’ desire for engagement, though it remains to be demonstrated whether these differences have clinical significance.

In addition to evaluating outcomes directly attributable to the DAs prior to the physician encounter, we also followed decision-making immediately after the physician encounter and assessed treatment received by chart review at 6 months. Patient–physician encounters were audio recorded, and we have previously published the qualitative findings. This is the first report to the findings from the survey data. Here, we show that treatment preferences varied by DA at the pre-encounter survey. The simple DA recipients expressed greater preference for watchful waiting (prior to meeting with their urologist). This result may be attributable to presentation of side effect rates in natural frequencies, and encouragement of shared decision making. There was also no difference by DA group in the treatment patients received at six months as assessed by medical record review [30].

One potential explanation for why patients’ earlier treatment preferences were not reflected in the treatment they ultimately received may be related to findings from our qualitative analysis of the conversations between patients and their clinicians (conducted with the current study population). In that analysis of data, we found that treatment received was based largely on urologists’ recommendations, which, in turn, were based on medical factors (age and Gleason score) and not on patients’ personal views of the relative pros and cons of treatment alternatives [30, 31]. Furthermore, we found that while physicians discussed treatment choice and risks and benefits in 95% of encounters, in more than one-third of encounters, physicians provided a partial set of treatment options and omitted surveillance as a choice. Additionally, while patient preferences were elicited in the majority of cases, they were not typically used to guide treatment planning. Thus, our analyses suggest that providing patients with DAs in preparation for the encounter may not produce treatment decisions that reflect their values for the outcomes potentially due to how physicians’ communicated about the treatment choices as well as a lack of inclusion of patient preferences. Our results spanning the entire decision process suggest more physician attention is needed to eliciting patient preferences and incorporating them in treatment decisions to accomplish informed and shared decision making. This process may be facilitated by incorporating a patient-centered comparative effectiveness table [60] with the benefits and harms computed objectively and specific to the patient's characteristics, so that patient and urologist are looking at the same chances for benefits and harms, and that these are accurate for each specific patient and by the physicians incorporating the patients values and goals into their discussions and recommendations of treatments.

### Limitations

Our study has several limitations. First, it was conducted in a Veteran population and the impact of a simple DA might differ in other populations. However, we improved the generalizability of our sample by recruiting patients from four regions of the United States. Furthermore, over a quarter of the sample had low health literacy. Second, our DAs differed in content, with one including information about advanced cancer, which could have influenced knowledge (the complex DA included this information, the simple aid did not). Pieterse et al. has found that inclusion of unnecessary information in decision aids can reduce knowledge of key facts [61]. Other content differences occurred as well, including use of tables in the plain language DA that were not replicated in the higher literacy DA. Third, several outcome measures (i.e., treatment received, perception of patient–physician communication) may be subject to contamination bias in that the provider would have interacted with patients receiving both types of aids. However, overall, our data show that physicians were not influenced by DAs. Fourth, all participants received the DA *before* getting their diagnosis. The impact of the decision aid might be different when patients receive the decision aid after their diagnosis. We deliberately delivered the DA prior to diagnosis because we believed patients would benefit from receiving information before they talked with their doctor. We also believed that receiving a DA earlier in the process might have a greater impact on treatment choices because patients would read the DA to determine their treatment preferences rather than to confirm the treatment decided on during the clinic visit. We note that there was attrition across the three time points within the study, although there were no significant differences between groups in terms of attrition. There may be differences in response to the decision aids between those who continued in the study and those who dropped out. While our study contributes to methods to support patient-centered care and patient involvement in decision making, we did not address cost or cost-effectiveness of DA production.

## Conclusion

This study suggests that decision aids may be necessary but not sufficient to affect the treatment patients receive. This conclusion is supported by a recent systematic review showing that knowledge alone was not enough for patients to be able to successfully engage in shared decision making with their physicians [62]. Rather, decision aids might more explicitly prepare patients to take a more active role in decision making. However, institutional support from health systems and provider organizations in the form of guidelines and quality of care measures that reward shared decision-making will be important.

## Supplementary Information

**Additional file 1.** Simple D. This is the full patient DA used in the study. This aid was developed by the authors and was not taken from another source.

**Additional file 2.** Complex DA. This is the medical DA used in the study.

**Additional file 3.** CONSORT checklist. This is the completed CONSORT checklist for the study.

## Data Availability

The datasets generated during and/or analyzed during the current study are available in the Clinical Trials repository: NCT00432601, Registered 8 February 2007 (prior to data collection): https://clinicaltrials.gov/ct2/show/results/NCT00432601?term=decision+aid&cond=Prostate+Cancer&draw=2&rank=6.

## Abbreviation

DA: Decision aid
