# Supplementary material for: Head to head randomized trial of two decision aids for prostate cancer
Source: BMC Med Inform Decis Mak. 2021 May 12;21:154. doi: 10.1186/s12911-021-01505-x (PMC8117645; doi:10.1186/s12911-021-01505-x)
Supplement: Supplementary file 2 — Additional file 2. Complex DA. This is the medical DA used in the study. [file 12911_2021_1505_MOESM2_ESM.pdf]

Prostate Cancer

Treatment Guidelines for Patients

Version VI/ October 2007

American Cancer Society®

NCCN National Comprehensive Cancer Network

Prostate Cancer

Treatment Guidelines for Patients

Version VI/ October 2007

The mutual goal of the National Comprehensive Cancer Network® (NCCN®) and the American Cancer Society (ACS) partnership is to provide patients and the general public with state-of-the-art cancer treatment information in an easy-to-understand language. This information, based on the NCCN's Clinical Practice Guidelines, is intended to assist you in a discussion with your doctor. These guidelines do not replace the expertise and clinical judgment of your doctor.

NCCN Clinical Practice Guidelines are developed by a diverse panel of experts. The guidelines are a statement of consensus of its authors regarding the scientific evidence and their views of currently accepted approaches to treatment. The NCCN guidelines are updated as new information becomes available. The Patient Information version is updated accordingly and is available on-line through the American Cancer Society and NCCN Web sites. To ensure you have the most recent version, contact the American Cancer Society at 1-800-ACS-2345 or the NCCN at 1-888-909-NCCN.

©2007 by the American Cancer Society (ACS) and the National Comprehensive Cancer Network (NCCN). All rights reserved. The information herein may not be reprinted in any form for commercial purposes without the expressed written permission of the ACS. Single copies of each page may be reproduced for personal and non-commercial uses by the reader.

Contents

Introduction 5
Making Decisions About Prostate Cancer Treatment 5
Inside and Around the Prostate 6
Detecting Prostate Cancer 7
Diagnosing Prostate Cancer 8
Prostate Cancer Stages 12
Types of Treatments for Prostate Cancer 15
Treatment of Pain and Other Symptoms 21
Complementary and Alternative Therapies 21
Side Effects of Prostate Cancer Treatments 22
Considering Treatment Options 25
Other Things to Consider 26
During and After Treatment 26
About Clinical Trials 27
Work-up (Evaluation) and Treatment Guidelines 31
Decision Trees
Prostate Cancer Work-up (Evaluation) 32
Initial Treatment for Prostate Cancer With Low to Intermediate Recurrence Risk 36
Initial Treatment for Prostate Cancer With High to Very High Recurrence Risk or Spread to Lymph Nodes or Distant Sites 40
Follow-up Surveillance and Management of Recurrence 44
Work-up and Treatment for Prostate Cancer That Returns After Radical Prostatectomy 48
Work-up and Treatment for Prostate Cancer That Returns After Radiation Therapy 50
Systemic Treatment for Widespread Prostate Cancer 52
Glossary 56

NCCN National Comprehensive Cancer Network

Member Institutions

Arthur G. James Cancer Hospital and Richard J. Solove Research Institute at The Ohio State University

City of Hope

Dana-Farber/Brigham and Women's Cancer Center Massachusetts General Hospital Cancer Center

Duke Comprehensive Cancer Center

Fox Chase Cancer Center

Fred Hutchinson Cancer Research Center/ Seattle Cancer Care Alliance

H. Lee Moffitt Cancer Center & Research Institute at the University of South Florida

Huntsman Cancer Institute at the University of Utah

Memorial Sloan-Kettering Cancer Center

Robert H. Lurie Comprehensive Cancer Center of Northwestern University

Roswell Park Cancer Institute

The Sidney Kimmel Comprehensive Cancer Center at Johns Hopkins

Siteman Cancer Center at Barnes-Jewish Hospital and Washington University School of Medicine

St. Jude Children's Research Hospital/ University of Tennessee Cancer Institute

Stanford Comprehensive Cancer Center

University of Alabama at Birmingham Comprehensive Cancer Center

UCSF Comprehensive Cancer Center

University of Michigan Comprehensive Cancer Center

UNMC/Eppley Cancer Center at The Nebraska Medical Center

The University of Texas M.D. Anderson Cancer Center

Vanderbilt-Ingram Cancer Center

Introduction

With this booklet, men with prostate cancer have access to information on the way prostate cancer is treated at the nation's leading cancer centers. Originally developed for cancer specialists by the National Comprehensive Cancer Network (NCCN), these treatment guidelines have now been translated for the lay public by the American Cancer Society (ACS).

Since 1995, doctors have looked to the NCCN for guidance on the highest quality, most effective advice on treating cancer. For more than 90 years, the public has relied on the American Cancer Society for information about cancer. The Society's books, brochures, and Web site provide comprehensive, current, and understandable information to hundreds of thousands of patients, their families, and friends. This collaboration between the NCCN and ACS provides an authoritative and understandable source of cancer treatment information for the public.

These patient guidelines will help you better understand your cancer treatment and your doctor's counsel. We urge you to discuss them with your doctor. You might begin by asking the following questions:

- How do my age, general health, and other medical conditions influence my treatment choices?
- What are the chances that my cancer can be treated successfully?
- What stage is my cancer and how does it influence my treatment options?
- How do the Gleason score of my cancer and my blood prostate-specific antigen (PSA) level predict my outlook for survival and affect treatment options?
- What do you feel my strongest treatment options are?
- Do you recommend one treatment over the others? If so, why?
- What are the likely side effects of each proposed treatment, and how will they affect my quality of life?
- What can be done to help manage the side effects of treatment?
- If I want to consult with other doctors, who would you recommend?
- How much time do I have before I need to decide on a particular treatment?

Making Decisions About Prostate Cancer Treatment

Prostate cancer is the most common cancer (excluding skin cancer) of American men. Most prostate cancers develop in older men and grow very slowly. But some can affect younger men, especially African Americans. African-American men are more likely to develop prostate cancer, and at a younger age, than other men. In some men, the cancer can grow quickly and spread to other parts of the body, causing symptoms and, sometimes, death. This too is more likely to happen to African-American men. Treating men with prostate cancer can help them live longer and can prevent or relieve symptoms. But treatment is not the right thing for all men with prostate cancer. One reason prostate cancer is so confusing to both doctors and patients is that it is difficult to tell which men will benefit from treatment and which will have side effects of treatment that will outweigh the benefits.

Prostate cancer is a disease that needs a team of doctors — often your primary physician, a surgeon *(urologist)*, radiation oncologist, and medical oncologist — to treat the disease. But not all men with prostate cancer should receive the same treatment, and, in some cases, the best treatment may be no treatment.

This booklet can help you and your doctor decide which choices best meet your medical and personal needs. You'll find flow charts that doctors call Decision Trees. These charts show how you and your doctor can arrive at the choices you need to make about your treatment.

To reach an informed decision you need to understand some of the medical terms your doctor uses. You may feel you're on familiar ground already, or perhaps you need to refer to the various sections listed in the Contents. Not only will you find background information on prostate cancer, but also explanations of cancer stages, work-up, and treatment. We've also provided a glossary at the end of the booklet. Words in the glossary appear in italics when first mentioned in this booklet.

Inside and Around the Prostate

The prostate is a *gland* found only in men. It is about the size of a walnut and is located in front of the rectum, internally, just behind the base of the penis. The prostate surrounds the internal part of the urethra, the tube that carries urine and semen out of the penis.

The function of the prostate gland is to produce some of the seminal fluid (or semen), which protects and nourishes sperm cells. The cells that make up the prostate gland grow and stay healthy because of the influence of the main male *hormone*, *testosterone*. A general term for all male hormones is *androgens*.

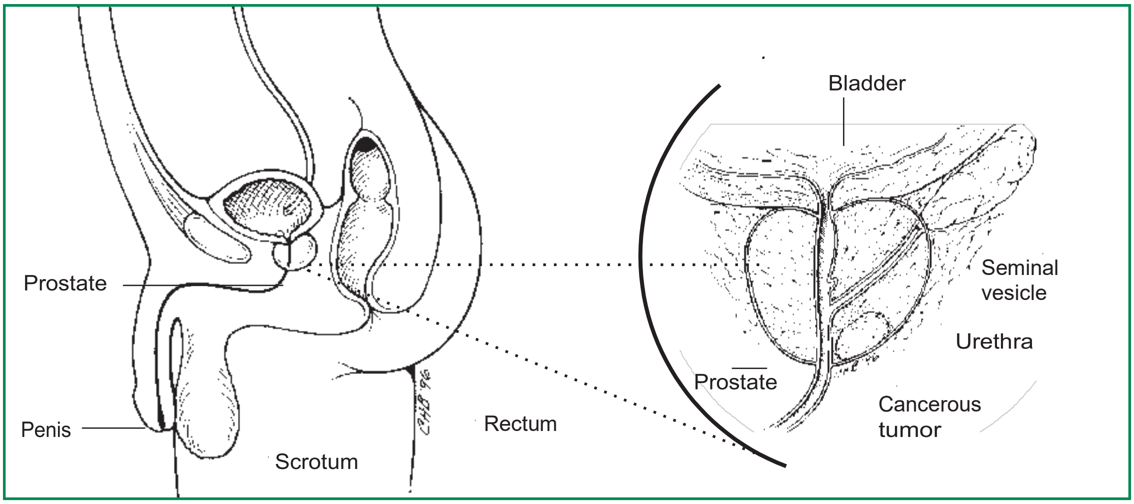

Figure 1. Location of the prostate gland

Prostate cancer develops from cells of the prostate gland. Almost all prostate cancers are adenocarcinomas, meaning that they develop from glandular cells. Prostate cancer generally grows slowly within the gland, but as it grows, it can eventually penetrate the outer rim of the gland. When this happens it may spread directly to tissues and organs near the prostate gland. Eventually the cancer cells may spread (metastasize) to distant parts of the body, particularly the bones.

If it spreads, prostate cancer tends to first go through lymphatic vessels to nearby *lymph nodes* in the pelvis. Lymph is a clear fluid that contains tissue waste products and immune system cells. Lymphatic vessels carry this fluid to lymph nodes (small, bean-shaped collections of immune system cells that are important in fighting infections). Cancer cells may enter lymphatic vessels and spread out along these vessels toward the lymph nodes, where they can continue to grow. If prostate cancer cells have reached the pelvic lymph nodes, it is likely they have spread to other organs of the body, too.

Surrounding the prostate gland are bundles of nerves and blood vessels. The nerves that run along the outside of the prostate gland help cause an erection of the penis. Treatments that destroy or damage these nerves can cause erectile dysfunction, also known as *impotence*.

Detecting Prostate Cancer

Many prostate cancers are found by *digital rectal examination (DRE)* and/or *prostate-specific antigen (PSA)* testing.

Prostate-Specific Antigen Blood Test

Prostate-specific antigen (PSA) is a substance made by the normal prostate gland. Most of the PSA is in semen and normally only a small amount escapes into the blood. Most men have levels under 4 nanograms per milliliter (ng/ml) of blood. But when prostate cancer develops, the PSA level usually goes above 4. Still, about 15% of men with a PSA below 4 will have prostate cancer when a *biopsy* is done. A man with a PSA level in the borderline range between 4 and 10 has about a 1 in 4 chance of having prostate cancer. If the PSA level is more than 10, the chance of having prostate cancer is over 50% and increases more as the PSA level increases. PSA levels estimate how likely a man is to have prostate cancer, but the test does not provide a definite answer. The diagnosis of prostate cancer can only be made by removing a sample of prostate tissue for examination (biopsy).

PSA is used as an *early detection* test for prostate cancer. It is also used to follow the status of the cancer in someone who has been treated. PSA is also made by the small intestine, breast, and salivary and parotid glands, so sensitive tests may detect PSA levels in very low levels (0.01–0.05). However, after surgery to remove the entire prostate the PSA level should drop to near 0 and levels should remain very low or even undetectable. If it doesn't completely fall to near 0, cancer may still be there. Or if it does drop to near 0 and then rises, it is likely the cancer has come back. After radiation therapy, the cancer usually falls to a low level but not 0. Once again if it begins to rise from this low point, then it is likely the cancer is growing back.

The PSA test can also help predict *prognosis* (outlook for survival). Men with very high PSA levels are more likely to have cancer that has spread beyond the prostate and are less likely to be cured or have long survival.

PSA levels can be used with clinical examination results and the tumor's Gleason score (discussed on page 9) to help determine which tests are needed for further evaluation and decide which is the best treatment option.

Digital Rectal Exam

During this examination, also known as DRE, a doctor inserts a gloved, lubricated finger into the man's rectum to feel the prostate for any irregular or abnormally firm area that might be a cancer. The prostate gland is located directly in front of the rectum. Most prostate cancers begin in the part of the gland that is nearest the rectum and can be reached by a rectal exam.

This exam is also used once a man is known to have prostate cancer in order to help predict whether the cancer has spread beyond his prostate gland.

Diagnosing Prostate Cancer

History and Physical Exam

When your doctor "takes a history," he or she will ask you a series of questions about your symptoms and risk factors. Most early prostate cancers cause no symptoms and are found by early detection testing. Advanced prostate cancers may be found because of symptoms such as slowing or weakening of the urinary stream or the need to urinate more often.

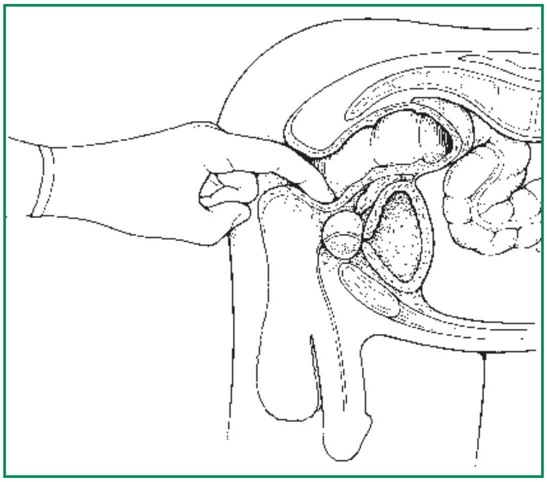

Figure 2. Digital rectal exam

These symptoms can also be caused by *benign prostatic enlargement (BPE)*. Other symptoms of advanced prostate cancer may include blood in the urine, swollen lymph nodes in the groin area, impotence (difficulty having an erection), and pain in the pelvis, spine, hips, or ribs. These symptoms may also be caused by other diseases and do not always mean that a man has prostate cancer. A physical exam to look for prostate cancer will include a DRE of the prostate. A general physical exam is also important in helping to detect or evaluate any other medical problems.

Transrectal Ultrasound and Biopsy

Transrectal ultrasound (TRUS) uses sound waves to create a picture of the prostate on a video screen. Sound waves are released from a small probe placed in the rectum. The same probe detects the echoes that bounce back from the prostate tissue, and a computer translates the pattern of echoes into a picture.

Less Common Types of Prostate Cancer

Most of the time, prostate cancer develops from the glandular tissue. But sometimes it starts in cells called neuroendocrine cells. This type of cell can have several appearances. One type is referred to as *small cell prostate carcinoma* (cancer). Otherwise it is just called *neuroendocrine prostate cancer*. This distinction is important because neuroendocrine cancers respond differently to treatment than the more common glandular prostate cancers do (See Chemotherapy on page 19).

If the doctor suspects prostate cancer because of the results of early detection tests or because of certain symptoms (such as blood in the urine, difficult urination, or pelvic pain), a biopsy of prostate tissue will be recommended to determine if the disease is present. **A biopsy is the only way prostate cancer can be diagnosed.**

A *core needle biopsy* is the main method used to diagnose prostate cancer. The doctor will use TRUS for guidance and place a narrow needle through the wall of the rectum into the prostate gland. The needle removes a cylinder of tissue, usually about one-half inch long and one-sixteenth inch across. This tissue is sent to the lab and examined under a microscope to see if cancer is present. The procedure is usually done in the doctor's office or outpatient clinic and takes less than half an hour. Though the procedure sounds painful, it typically causes little discomfort because a special instrument, called a biopsy gun, inserts and removes the needle in a fraction of a second. The doctor also may numb the area with a local anesthetic.

If the TRUS doesn't show a tumor, biopsy samples are taken from many different areas of the prostate. Usually 6 to 12 cores are removed (from upper, mid, and lower areas of the left and right sides) to get a good sample of the gland and tell how much of the gland (if any) is affected by the cancer.

If the doctor looking at the biopsy under the microscope (a *pathologist*) believes it looks suspicious, meaning that some cells do not look normal but are not clearly cancerous, the biopsy may be repeated and may include more samples of the prostate.

Cancer Grade or Gleason Score

If a cancer is found, it will be graded to estimate how aggressive it is likely to be. The tissue samples taken during the prostate biopsy are examined and graded according to how closely they look like normal prostate tissue when viewed under a microscope.

The most commonly used prostate cancer grading system is called the *Gleason system*. This system assigns 2 grades, a primary and secondary grade. Each grade ranges from 1 through 5 based on the ability of the cancer cells to form glands. If the cells are arranged in clusters that look like the glands of normal prostate tissue, a Gleason grade of 1 is assigned. If the cancer lacks these features and its cells seem to spread unevenly through the prostate, it is a grade 5 tumor. Grades 2 through 4 have intermediate features.

If you have questions about your biopsy results, the pathology report, or any other part of the diagnostic process, do not hesitate to ask your doctor. You can get a pathology review by having your tissue sample (specimen) sent to a consulting pathologist at an NCCN center or a laboratory recommended by your doctor.

Because prostate cancers often have areas with different grades, 2 grades are given. There is a primary (most common pattern) grade and a secondary (second most common pattern) grade. These grades are assigned to the 2 areas that make up most of the cancer. The Gleason primary and secondary grades are added (e.g., 3 + 2 = 5) to yield the Gleason score (range 2–10). The higher the score, the more likely it is that the cancer will grow and spread quickly.

Lymph Node Biopsy

The purpose of this test is to find out if cancer has spread from the prostate to nearby lymph nodes. This is only done before surgery or radiation therapy if a computed tomography (CT) scan or magnetic resonance imaging (MRI) (see pages 11–12) shows enlarged lymph nodes.

If lymph nodes look enlarged on the imaging study, a specially trained radiologist may take a sample of cells from a lymph node by using a technique called *fine needle aspiration (FNA)*. In this procedure, the doctor uses a CT scan image to guide a long, thin needle into the lymph node. A syringe attached to the needle is used to take a small tissue sample. If cancer cells are found in the lymph node biopsy specimen, surgery is usually not attempted. Instead, other treatment options are considered because the cancer has probably spread to other areas too. There are several other ways lymph node biopsies are done.

During prostate surgery, the surgeon may remove lymph nodes through an incision in the lower part of the abdomen (belly). This is often done in the same operation as the planned *radical prostatectomy* (discussed later). If the lymph nodes contain cancer, the prostate may not be removed and further treatment may be given.

In some cases, the surgeon may use a *laparoscope*, which is a long, thin, flexible tube inserted into the abdomen through a very small incision. Using one or more other small incisions, the surgeon can remove the lymph nodes around the prostate gland with special surgical instruments and send them to the pathologist. This procedure (laparoscopic lymphadenectomy) is rarely used.

Blood Tests

A complete blood count (CBC) determines whether the patient's blood has the correct number of various types of blood cells. Abnormal test results may suggest spread of cancer to the bone marrow, where blood cells are made. Doctors repeat this test regularly in patients treated with *chemotherapy* because these drugs temporarily affect the blood-forming cells of the bone marrow.

If prostate cancer spreads to the bones it may cause certain chemical abnormalities in the blood. To detect these changes, doctors look at blood chemistry tests for certain substances, such as *alkaline phosphatase*. Levels of this

enzyme often go up in men whose prostate cancer has spread to the bones or liver.

Some of the drugs used in *hormone therapy* can interfere with liver function. If the cancer spreads to the liver, that can affect liver function, too. These changes in liver function can also be detected by blood tests.

Imaging Tests

These tests use x-rays, magnetic fields, or radioactive substances to create pictures of the inside of the body to look at the extent of spread of the cancer. Several types of imaging tests may be used to look for cancer that has spread beyond the prostate gland, but none of the tests is perfect. Imaging tests are done if early tests, such as the DRE and PSA, and the Gleason score from the prostate biopsy indicate that the cancer is likely to have spread.

Radionuclide bone scan

When prostate cancer spreads to distant sites it often goes to the bone. A bone scan is a test that shows whether the cancer has spread from the prostate gland to bones. For this test you will get an injection of a small amount of radioactive material called technetium diphosphonate. The amount of radioactivity used is very low and causes no long-term effects. The radioactive substance is attracted to diseased bone cells throughout the entire skeleton over the course of a couple of hours. A special camera then detects the radioactivity and creates a picture of your skeleton. Any areas of diseased bone will be seen on the bone scan image as dense, gray to black areas called "hot spots." These areas may suggest the presence of metastatic cancer, but arthritis, infection, or other bone diseases can cause similar patterns.

A bone scan is not routinely done before prostate cancer treatment unless there are signs of aggressive disease, such as a significantly elevated PSA level, a high Gleason score, or symptoms that could be caused by cancer.

Computed tomography

Commonly referred to as a *CT* or *CAT scan*, this test uses a rotating x-ray beam to produce detailed cross-sectional images of your body. Instead of taking one picture, as does a usual chest x-ray, a CT scanner takes many pictures as it rotates around you. A computer then combines these pictures into an image of a slice of your body. The machine will take pictures of multiple slices of the part of your body that is being studied.

Often after the first set of pictures is taken, you may be asked to drink 1 or 2 pints of a radio contrast agent or "dye," or you may receive an intravenous injection of a radiocontrast agent or dye. This helps to better outline structures in your body. You will also need to drink enough liquid to have a full bladder. This will keep the bowel away from the area of the prostate gland. A second set of pictures is then taken. Some people feel flushed or get hives or, rarely, more serious allergic reactions like trouble breathing and low blood pressure can occur. Be sure to tell the doctor if you have ever had a reaction to any contrast material used for x-rays.

The CT scan will give exact information about the size, shape, and position of a tumor and may help find enlarged pelvic lymph nodes that might contain cancer. The CT scan can also detect cancer that has spread to other internal organs, such as the liver.

A CT scan is usually not done to evaluate early *stage* disease before treatment, unless there is a 7% or more chance that lymph nodes are involved. Your doctor can determine this by checking the information about your cancer with tables called the *Partin tables* (discussed on page 15).

Magnetic resonance imaging

MRI scans take pictures using radio waves and strong magnets instead of x-rays. The energy from the radio waves is absorbed and then released in a pattern formed by the type of tissue and by certain diseases. A computer translates the pattern of radio waves given off by the tissues into very detailed cross-sectional images of parts of the body. A contrast material might be injected, just as with CT scans. To improve the accuracy of the MRI, many doctors will place a probe, called an endorectal coil, inside your rectum. This must stay in place for 30 to 45 minutes and can be uncomfortable.

MRI pictures can show abnormal lymph nodes or changes in internal organs that suggest cancer may have spread. As with the CT scan, an MRI scan is usually not done to evaluate early stage disease before treatment unless there is reason to believe the lymph nodes may have cancer cells in them.

ProstaScint® scan

The ProstaScint® scan uses low-level radioactive material to find cancer that has spread beyond the prostate. The radioactive material for the ProstaScint® scan is attached to a monoclonal antibody, a type of antibody made in the lab to recognize and stick to a particular substance. In this case, the antibody specifically recognizes prostate-specific membrane antigen (PSMA), a substance found only in normal and cancerous prostate cells. After the material is injected, you will be asked to lie on a table while a special camera creates an image of the body. This is usually done about half an hour after the injection and again 3 to 5 days later.

The advantage of this test is that it has the potential to detect prostate cancer that has spread to bone, lymph nodes, and other organs and may distinguish prostate cancer from other cancers and benign disorders. The disadvantage is the lack of specificity, meaning that it often suggests spread when there is none. The ProstaScint® scan is usually not used to stage the cancer before initial treatment. It may prove to be more useful after treatment in cases where it is thought that the cancer has come back (recurred).

Prostate Cancer Stages

A prostate cancer's stage indicates how far it has spread within the prostate, to nearby tissues, and to other organs. The stage of a cancer is one of the most important factors in selecting treatment options. It is also the most significant (but not the only) factor in predicting a man's outlook for survival (prognosis).

A staging system is a standardized way in which the cancer care team describes the extent to which a cancer has spread. The most commonly used system in the United States is called the TNM System of the American Joint Committee on Cancer (AJCC). The TNM System describes the extent of the primary tumor (T), the absence or presence of spread

to nearby lymph nodes (N), and the absence or presence of spread to distant organs (M).

The stages described here are based on the most recent version (2002) of the AJCC staging manual.

T Categories

There are actually two types of T classifications for prostate cancer.

- The *clinical stage* is your doctor's best estimate of the extent of the disease based on digital rectal exam (DRE), needle biopsy, and any imaging studies that were done.
- The *pathologic stage* is based on surgical removal and examination of the entire prostate gland, both *seminal vesicles* (two small sacs next to the prostate that store semen) and, in some cases, nearby lymph nodes.

The clinical stage is used in making treatment decisions, such as whether a patient might benefit from treating the prostate cancer with surgery or radiation. However, the clinical stage may underestimate the extent of cancer spread, and if surgery is done, the pathologic stage assigned after surgery is more accurate. Men who do not have a radical prostatectomy (surgery to remove the prostate gland, seminal vesicles, and nearby tissues) do not have a pathologic T stage determined. There are 4 categories for describing the prostate cancer's T stage.

T1 refers to a **tumor** that is not felt during a digital rectal exam, but cancer cells are found in a prostate biopsy or prostatectomy specimen (if prostatectomy has been performed for benign prostatic enlargement). T1 prostate cancers can be further subclassified as T1a, T1b, and T1c.

- **T1a** describes prostate cancers found incidentally (by accident) during *transurethral resection of the prostate (TURP)* or open prostate removal (simple prostatectomy). These are surgical procedures done to relieve symptoms of benign prostate hypertrophy (prostate enlargement that is not caused by cancer). This operation is usually done because the enlarged prostate gland presses on the urethra and makes it difficult for a man to urinate. When prostate tissue is removed and checked under the microscope, cancer may be found, even though the doctor who removed the tissue did not expect cancer to be present. T1a indicates that less than 5% of the tissue removed is cancer and more than 95% is benign.
- **T1b** also describes cancers found incidentally during TURP or simple prostatectomy, but more than 5% of the removed tissue is cancer.
- **T1c** cancers are found by biopsy. In these cases a core needle biopsy is usually done because the PSA blood test result was elevated, suggesting that a cancer might be present.

T2 means that a doctor can feel the prostate cancer by DRE and that the cancer is thought to remain within the prostate gland. This category is subclassified into T2a and T2b and T2c.

- **T2a** means that the cancer is in only one side of the prostate, and is in only half (or less) of that side.

- **T2b** cancers are in only one side of the prostate, but are in more than half of that side.
- In **T2c** cancer, the cancer is in both sides of the prostate gland.

T3 cancers have spread beyond the outer rim *(capsule)* that surrounds the gland. They have reached the connective tissue next to the prostate and/or the seminal vesicles, but have not spread to any other organs. This group is divided into T3a and T3b.

- In **T3a**, the cancer is growing outside the prostate but has not spread to the seminal vesicles.
- A **T3b** cancer has spread to the seminal vesicles.

T4 means that the cancer has spread to tissues next to the prostate (other than the seminal vesicles), such as the bladder neck or its external sphincter (muscle that helps control urination), the rectum, the muscles in the pelvis, or the wall of the pelvis.

N Categories

The N category is determined by whether or not the cancer has been found in nearby lymph **nodes**.

NX means that tests to detect lymph node spread have not been done.

N0 means that the cancer has not spread to any lymph nodes.

N1 means spread to nearby lymph nodes in the pelvis

M Categories

The M category stands for **metastasis**, or whether or not the cancer has spread to distant organs.

MX means that tests to detect distant spread have not been done.

M0 means that the cancer has not spread beyond the nodes in the pelvis.

M1 means the cancer has spread to distant sites.

- **M1a** means the cancer has spread to distant lymph nodes.
- **M1b** means the cancer has spread to bone(s).
- **M1c** means the cancer has spread to other organs such as lungs, liver, or brain.

Although in this AJCC staging system, the T, N, M stages are to be combined into a single Roman numeral I–IV, this is not often needed for prostate cancer because the stages follow the T stage. Stage I = T1, Stage II = T2, Stage III = T3, and Stage IV includes T4 and spread to lymph nodes or distant sites.

A special note about bone metastases

Most of the time even though the cancer has spread to the bone, the bone is not weakened. X-rays often show that the bone appears denser and even harder. These are called *blastic* metastases. Sometimes, the cancer will dissolve the bones and severely weaken them. These are called *lytic* metastases. Lytic metastases may be caused by the small cell or neuroendocrine type of prostate cancer.

Partin tables

These tables take the results of the PSA test, the clinical stage (T1 or T2), and the Gleason score and combine them to predict the chance that the cancer has spread outside the prostate, to the seminal vesicles, or to nearby lymph nodes. The tables are used to help estimate a patient's risk of spread so that appropriate tests, such as CT scan or MRI, may be done. They also help doctors plan treatment. The tables have not been included in this booklet but can be found on the Web at http://urology.jhu.edu/Partin_tables/.

Some of the treatment options listed in the patient Decision Trees are based on the probability of cancer spread. These probabilities come from Partin Tables.

Types of Treatments for Prostate Cancer

Depending on the stage of their disease, men often have more than one treatment option to consider. Several factors should be taken into account when choosing between these options, including both the potential benefits and risks. The side effects associated with each of type of prostate cancer treatment are discussed in the next section.

Radical Prostatectomy

In a radical prostatectomy, the surgeon removes the entire prostate gland plus some tissue around it. This operation is used most often if the cancer appears not to have spread outside the gland. During the surgery the patient is either under general anesthesia (asleep and totally unconscious) or under spinal or epidural anesthesia (the same type of anesthesia often given to women during childbirth to numb the lower half of the body) with sedation.

There are 2 main types of radical prostatectomy: radical retropubic prostatectomy and radical perineal prostatectomy. In the retropubic operation, the surgeon makes a skin incision in the lower abdomen. The surgeon may remove lymph nodes during this operation through the same incision. A nerve-sparing radical retropubic prostatectomy is a modification of this operation. During this procedure, the surgeon carefully examines the small bundles of nerves on either side of the prostate gland. If it appears that the cancer has not spread to these nerves, the surgeon will try to not remove or damage them. Because these are the nerves that are needed for erections, leaving them intact lowers (but does not eliminate) the risk of impotence (not being able to have an erection) following surgery.

A newer approach to surgery is laparoscopic radical prostatectomy (LRP). The surgery begins with the urologist making several small incisions in the patient's abdomen. (The incisions are one-fourth to one-half inch long, compared to a single 5- to 6-inch long incision for traditional surgery.) A laparoscope – a long, thin, lighted video camera – is then inserted through one of the incisions. Tiny

surgical instruments are inserted into the other incisions. The surgeon sometimes uses a robotic system (called the da Vinci system) to control the movement of the instruments. Mini-cameras on the instruments send images to video monitors. These images are larger than life, magnified many times, allowing the surgery to be extremely precise. The magnified view also helps the surgeon avoid damaging the delicate structures and nerves surrounding the prostate. LRP has been used in the United States since 1999. Robotic LRP was developed in the United States in 2000. Both are more frequently being done in university centers, are relatively new procedures, and require skilled and experienced surgeons.

In the radical perineal prostatectomy, the prostate is removed through an incision in the skin between the scrotum and anus. Nerve-sparing operations are more difficult with this approach, and lymph nodes cannot be removed through this incision. If men having a radical perineal prostatectomy need lymph nodes examined, the surgeon can remove some nodes through a very small skin incision in the abdomen or by using a laparoscope (discussed earlier).

Open operations are followed by an average hospital stay of 2–3 days and the average time away from work is 3 to 6 weeks. A *catheter* (a thin, flexible tube) is usually inserted through the penis and into the bladder after surgery while the patient is still asleep. The catheter is kept in place for 7 to 10 days to help patients urinate easily while they heal. With the laparoscopic prostatectomy, patients generally go home the day after surgery.

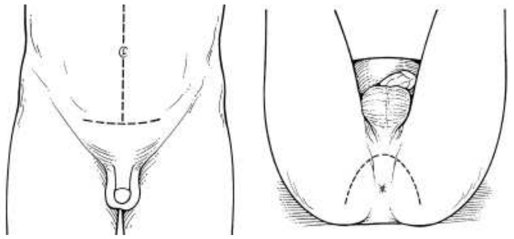

Figure 3. Radical prostatectomy

Radiation Therapy

Radiation therapy (RT) uses high-energy rays (such as x-rays) or particles (such as electrons or protons) to kill cancer cells. Radiation is sometimes used to treat prostate cancer that is still confined to the prostate gland or has spread to nearby tissue. If the disease is more advanced, radiation may be used to reduce the size of the tumor or to provide pain relief when cancer has spread to the bones. The 2 main types of radiation therapy are *external beam radiation* and *brachytherapy* (internal radiation).

External beam radiation

External beam radiation is focused from a source outside the body on the area affected by the cancer. It is much like getting a diagnostic x-ray but for a longer time. Before treatments start, imaging studies are done to find the exact location of the cancer. The radiation team will then make ink marks on the patient's skin that they will use later as a guide for focusing the radiation in the right area. Patients are usually treated 5 days per week in an outpatient center over a period of 7 to 9 weeks. Each treatment lasts only a few minutes and is painless.

Three-dimensional conformal radiation therapy (3D-CRT) can more accurately target the prostate. This can reduce side effects, particularly damage to the rectum. It uses sophisticated computers to precisely map the location of the cancer within the prostate. The patient may be fitted with a plastic mold resembling a body cast to keep him still and in one position so that the radiation can be more accurately aimed. Since the prostate can move, it may be imaged daily so that the radiation beam targets it more precisely. Radiation beams are then aimed from several directions. Short-term results suggest that by aiming the radiation more accurately, it is possible to reduce radiation damage to tissues near the prostate and improve effectiveness by increasing the radiation dose to the cancer. For this reason, 3D-CRT is now the preferred method when using external beam radiation for the initial treatment of prostate cancer.

An advanced type of 3D-CRT is called intensity modulated radiation therapy (IMRT). IMRT can even more precisely map the location of cancer and focus high doses of radiation on it and not the surrounding normal tissue.

External beam radiation can also be used at specific sites to relieve bone pain from prostate cancer metastasis.

Internal radiation therapy (brachytherapy)

Internal radiation therapy uses small radioactive pellets (each about the size of a grain of rice) that are placed directly into the prostate. They can be left in place permanently or temporarily. Imaging tests such as transrectal ultrasound, CT scans, or MRI are used to accurately guide placement of the radioactive material into the cancer.

The permanent pellets are sometimes called seeds. The radioactive seeds (made of isotopes such as iodine-125 or palladium-103) are placed inside thin needles, which are inserted through the skin of the perineum (area between the scrotum and anus) into the prostate. The seeds are left in place as the needles are removed and give off low doses of radiation for weeks or months. Radiation from the seeds travels a very short distance, so the seeds can put out a very large amount of radiation to a very small area. This decreases the amount of damage done to the healthy tissues that are close to the prostate. Usually, anywhere from 40 to 100 seeds are placed. Because they are so small, their presence causes little discomfort. They are left in place after their radioactive material is used up. This type of radiation therapy requires spinal anesthesia (where the lower half of your body is numbed) or general anesthesia (where you are asleep) and may require 1 day in the hospital.

Alternatively, more radioactive material can be placed for less than a day. This approach is called high-dose rate (HDR) brachytherapy, and it is usually combined with low-dose external beam radiation. Hollow needles are placed through the perineum into the prostate. Soft nylon tubes (catheters) are placed in these needles. The needles are then removed but the catheters stay in place. Radioactive iridium-192 or cesium-137 is then placed in the catheters, usually for 5 to 15 minutes. Generally, about 3 brief treatments are given, and the radioactive substance is removed each time. The treatments are usually given over a couple of days.

After the last treatment the catheters are removed. For about a week following insertion of the needles, patients may have some pain in the perineal area and may have red-brown discoloration of their urine.

Systemic radiation therapy

Strontium-89 and samarium-153 are radioactive substances used to treat bone pain caused by metastatic prostate cancer. They are injected into a vein and settle in areas of bone that contain cancer. The radiation given off kills the cancer cells and relieves some of the pain caused by bone metastases. The majority of prostate cancer patients with painful bone metastases are helped by these treatments. If prostate cancer has spread to many bones, this approach is better than trying to aim external beam radiation at each affected bone.

Hormone Therapy (Androgen Deprivation Therapy)

Hormone therapy is often used for patients whose prostate cancer has spread beyond the prostate or has come back (recurred) after treatment. It can also be combined with radiation therapy for certain stage T3 cancers. The goal of hormone therapy is to block the effect of the male hormones, called androgens, which is why this treatment is often referred to as *androgen deprivation therapy* or ADT. The main androgens are testosterone and dihydrotestosterone (DHT). Androgens are produced mainly in the *testicles* and cause prostate cancer cells to grow. Lowering androgen levels or blocking their action can make prostate cancers shrink or grow more slowly. But hormone therapy alone does not cure the cancer.

Some prostate cancers do not respond to hormone therapy. These are called *androgen-independent cancers*. Often prostate cancers respond to hormone therapy for a few years before becoming androgen-independent. Less often, prostate cancers may be androgen-independent at the time they are diagnosed. Prostate cancer that starts off being androgen-independent may be a different kind of prostate cancer called small cell or neuroendocrine (See box, page 9).

There are several methods used for hormone therapy:

Orchiectomy

Orchiectomy is the removal of the testicles (male reproductive glands found in the scrotum). Although it is a surgical treatment, orchiectomy is considered hormone therapy because it works by removing the main source of male hormones. By lowering androgen levels, orchiectomy is able to shrink or slow the growth of most prostate cancers for a period of time.

Luteinizing hormone-releasing hormone agonists

These drugs can decrease the amount of testosterone produced by the testicles as effectively as surgically removing the testicles. *luteinizing hormone-releasing hormone (LHRH) agonists* are injected or placed as small implants under the skin either monthly or every 3, 4, or 12 months. The LHRH agonists currently available in the United States are goserelin, leuprolide, and triptorelin. All are

Table 1. Drugs Mentioned in this Guideline: Generic (Brand) Names

bicalutamide (Casodex®)	flutamide (Eulexin®)	nilutamide (Nilandron®)
carboplatin (Paraplatin®)	goserelin (Zoladex®)	pamidronate (Aredia®)
cisplatin (Platinol®)	ketoconazole (Nizoral®)	samarium-153 (Quadramet®)
docetaxel (Taxotere®)	leuprolide (Lupron®, Viadur®, Eligard®)	strontium-89 (Metastron®)
Diethylstilbestrol or DES	triptorelin (Trelstar®)	zoledronic acid (Zometa®)
etoposide (VP-16, VePesid®)		

about equally effective. They work by actually stimulating the pituitary gland to release hormones that cause testosterone production. After about 3 weeks, the pituitary gland "runs out" of these hormones and testosterone production drops.

Understanding this action is important because early in the treatment there can be a surge of testosterone production. This can cause the cancer to temporarily grow. If the cancer is in the bones, a patient may feel more bone pain. This is called a "flare" reaction. Flare often can be avoided by giving drugs called *anti-androgens* for a few weeks when starting treatment with LHRH agonists.

Anti-androgens

Anti-androgens block the body's ability to use androgens. Drugs of this type, such as bicalutamide, flutamide, and nilutamide, are taken as pills, up to 3 times a day. Anti-androgens can be used alone, but are often combined with LHRH agonists. This is called *combination hormone therapy*, or total androgen blockade. Combination hormone therapy probably offers no advantage over LHRH agonists or orchiectomy when used alone. These drugs are also used early in the course of treatment with LHRH agonists to block the flare reaction. Finally, anti-androgens may be tried when hormone therapy for advanced prostate cancer has failed. In this setting, anti-androgens are called second-line hormone therapy.

Other hormone drugs

The female hormone estrogen (diethylstilbesterol or DES) is sometimes effective after other hormone treatments have stopped working. Ketoconazole, initially used for treating fungal infections and later found to block androgen production, is another drug for second-line hormone therapy.

Chemotherapy

Chemotherapy or chemo is an option for patients whose prostate cancer has spread outside of the prostate gland and for whom hormone therapy is no longer working. It is not expected to destroy all of the cancer cells, but it may shrink the cancer or slow its growth and reduce pain.

Table 2. Expectant Management

A digital rectal exam (DRE) and prostate-specific antigen (PSA) blood test every 6-12 months, depending on life expectancy
A needle biopsy of the prostate gland within 6 months if fewer than 10 cores were taken at the first biopsy or if the tumor appeared on DRE to be on the side opposite the positive biopsy site
A repeat biopsy may be performed within 18 months if more than 10 biopsy cores were taken when the diagnosis was made and anytime thereafter if it looks like the cancer is growing

Chemotherapy uses anti-cancer drugs that are injected into a vein, injected into a muscle, or taken by mouth. These drugs kill cancer cells, but they also damage some normal cells. The doctor must maintain a delicate balance of chemo doses, making them high enough to kill the cancer cells, but not high enough to destroy too many healthy cells.

Docetaxel is a chemo drug that is used to treat prostate cancer that has returned or continued to grow and spread after treatment with hormone therapy. The NCCN experts state that the first chemo regimen a patient receives should include docetaxel. Docetaxel may be combined with other drugs to reduce the chances of the cancer cells becoming resistant to chemo.

Small cell prostate carcinoma or neuroendocrine cancer is a rare type of prostate cancer that is more likely to respond to chemotherapy than to hormone therapy. Small cell carcinoma develops most often in the lungs. Because small cell lung cancer often responds to chemotherapy with cisplatin and etoposide, these drugs are recommended for treating small cell cancers that develop in the prostate.

Expectant Management (Watchful Waiting or Observation)

One strategy for some patients with prostate cancer may be to "watch and wait" with no immediate active treatment. The cancer is regularly and carefully observed and monitored.

This approach may be recommended if a prostate cancer is not causing any symptoms (especially if it is very small and contained to one area of the prostate), if it is expected to grow very slowly, or if the patient is older or has other serious health problems. Because prostate cancer often grows very slowly, many older men who have the disease never need treatment. Some men may decide that the side effects of active treatment outweigh the benefits they hope to achieve. In these instances, the man may opt for expectant management (watchful waiting). Choosing expectant management does not mean that active treatment cannot be used if the cancer begins to grow more quickly or causes symptoms.

Treatment of Pain and Other Symptoms

Most of this document discusses ways to remove or destroy prostate cancer cells or to slow their growth. But it is important to realize that maintaining your quality of life is a very important goal. Don't hesitate to discuss your symptoms or any other quality-of-life concerns with your cancer care team.

As discussed before, radiation therapy (either external beam therapy or medicines such as strontium-89 or samarium-153) can be used to relieve bone pain caused by prostate cancer metastasis.

Bisphosphonates are a group of medicines that can be used to slow the damage caused by the spread of cancer within bones and prevent fractures. These drugs may relieve pain caused by bone metastases and may slow growth of these metastases. They also may have the added benefit of strengthening bones in men who are receiving hormone therapy. The bisphosphonates most commonly used are pamidronate and zoledronic acid. Both are given by intravenous (IV) injection, but the time of infusion is much shorter for zoledronic acid.

There are other effective and safe ways to treat pain, most other symptoms of prostate cancer, and most of the side effects caused by prostate cancer treatments. When properly prescribed, drugs can effectively relieve pain without risk of addiction, dependence, or becoming too drowsy to continue most of your usual activities. Enduring unnecessary pain has no benefit whatsoever. Pain medication does not interfere with anti-cancer treatments. In fact, getting effective pain relief can help some patients be more active and may, indirectly, help them live longer.

Complementary and Alternative Therapies

Complementary and alternative medicines are a group of different types of health care practices, systems, and products that are not part of your usual medical treatment. They may include Chinese herbs, special supplements, acupuncture, massage, and a host of other types of treatment.

The American Cancer Society defines complementary treatment methods as those that are used along with your regular medical care. Some methods that can be used in a complementary way are meditation to reduce stress, acupuncture to relieve pain, or peppermint tea to relieve nausea. There are many others. Some of these methods are known to help and may add to your comfort and well being, while others have not been tested. Some have been proven not to be helpful. A few have even been found harmful.

Alternative treatments are defined as those that are used instead of your regular medical care. These treatments have not been proven to be safe and effective in *clinical trials*. Some may even be dangerous or have life-threatening side effects. The most common danger is that you may lose the chance to be helped by standard treatment. Delays or interruptions in your standard medical treatment may give the cancer more time to grow.

Deciding What to Do: It is easy to see why people with cancer may consider alternative treatments. You want to do all you can to fight the cancer. Sometimes mainstream treatments such as chemo can be hard to take. And sometimes they stop working.

At times like this, when people suggest that their treatment can cure your cancer without serious side effects, it's normal to want to believe them. But the truth is that most non-standard treatments have not been tested and proven to be effective for treating cancer. As you consider your options, talk to your doctor or nurse about any treatment you are thinking about using. Call the American Cancer Society at 1-800-ACS-2345 or visit www.cancer.org to learn more about the specific treatments you are considering.

With reliable information and the support of your health care team, you may be able to safely use methods that can help you while avoiding those that could be harmful.

Side Effects of Prostate Cancer Treatments

Almost all prostate cancer treatments have side effects or unwanted effects caused by the treatment. For some men side effects are temporary, but they may last a long time for others. It is important that you discuss any problems or concerns with your health care team. They can help you ease, manage, or even prevent some side effects.

Incontinence

Incontinence is the inability to control the urine stream, resulting in leakage or dribbling of urine. It can affect you not only physically, but emotionally and socially, too.

Incontinence is divided into 3 types: stress incontinence, overflow incontinence, and urge incontinence.

- Stress incontinence causes urine leakage when coughing, laughing, sneezing, or exercising. It is usually caused by problems with the bladder sphincter (the muscular valve that keeps urine in the bladder). Prostate cancer treatments may damage the muscles that form this valve or the nerves that keep the muscles working. Stress incontinence is the most common type of incontinence after prostate surgery.
- Men with overflow incontinence take a long time to urinate and have a dribbling stream with little force. Overflow incontinence is usually caused by cancer or scar tissue blocking or narrowing the bladder outlet. This makes it difficult to empty the bladder.
- Men with urge incontinence often have a sudden need to go to the bathroom to pass urine *(urinary urgency)*. This problem occurs when the bladder becomes overly sensitive to stretching as urine fills it. Urge incontinence is the most common type of incontinence after radiation therapy and may occur after either external radiation or brachytherapy (seeds).

Treating incontinence depends on its type, cause, and severity. Some men feel embarrassed about discussing this issue, but it is important to remember that this is a common medical problem. Depending on your

situation, there are several ways to improve this condition, including surgery, medicine, and Kegel exercises which strengthen the muscles of the pelvis.

Impotence

Impotence, also known as erectile dysfunction (ED), is the inability to get an erection of the penis sufficient for sexual intercourse. The nerves that allow men to get erections are often damaged by radical prostatectomy or radiation therapy. Other treatments may also damage these nerves or the blood vessels that supply blood to the penis to cause an erection.

For men who are impotent, several solutions are available.

- Drugs such as sildenafil (Viagra®), vardenafil (Levitra®), or tadalafil (Cialis®) do not cause erections, but can improve erections by increasing blood flow to the penis.
- Vacuum devices can create an erection. These mechanical pumps are placed around the entire penis before intercourse.
- Prostaglandin E1 is a substance naturally made in the body that can produce erections. It can be injected almost painlessly into the base of the penis 5 to 10 minutes before intercourse or put into the urethra as a suppository.
- Prostheses (penile implants) can restore the ability to have erections.

Side Effects of Prostate Surgery

The main side effects of radical prostatectomy (RP) are incontinence and impotence.

Normal bladder control usually returns within several weeks or months after RP. Passing a small amount of urine when coughing, laughing, sneezing, or exercising may last a long time in up to 35% of men. Some patients (between 1% and 5%) have more serious stress incontinence, which may be permanent.

During the first 3 to 18 months after RP, most men will have erectile dysfunction and will need to use medicines or other treatments if they want to have an erection. The effect of this operation on a man's ability to achieve an erection is related to the patient's age, his ability to get an erection before the operation, and whether nerve-sparing surgery was done.

Nearly all men who have a RP should expect some permanent decrease in their ability to have an erection, but younger men may expect to retain more of their ability. If the surgeon does not remove the nerves on either side of the prostate during prostatectomy, the impotence rate is between 25% and 30% for men under 60. But it occurs in 70% to 80% of men over 70, even if nerves on both sides are not removed.

Incontinence and impotence are reported less often among men treated at major cancer centers, where this type of surgery is performed on a more routine basis.

Side Effects of Radiation Therapy

The main side effects of radiation therapy for prostate cancer are injury to the bladder and rectum and impotence (inability to get an erection).

Both during and after treatment, side effects may include frequent urination, urge

incontinence (feeling like you have to urinate all the time), burning sensation while urinating, and blood in the urine. Less than 5% of men report problems with urinary incontinence in the first 5 years after treatment. But this may increase over time because the chances of having side effects after radiation go up each year after treatment. Side effects of external beam radiation may include diarrhea, blood in the stool, and colitis (irritated intestines). Occasionally, normal bowel function does not return after treatment is stopped. The newer conformal radiation techniques may be less likely to cause bladder and bowel side effects.

About 30% to 70% of men who receive external beam radiation develop impotence. Impotence usually does not occur right after radiation therapy, but gradually develops over one or more years. The long term rate of impotence is similar to that associated with surgery. As with surgery, the older the man is, the more likely it is he will become impotent. Impotence may be helped by treatments such as those listed earlier, including erectile dysfunction medicines.

Brachytherapy may also result in impotence, urinary incontinence, and bowel problems. Significant rectal problems (burning, pain, and diarrhea) may occur in approximately 5% of patients and are difficult to treat once they develop. While about one-third of men may have frequent urination, severe incontinence is not common. But as with external beam radiation, side effects may worsen several years after treatment. Impotence may be less likely to develop after brachytherapy than external radiation initially, but this problem also can increase over time.

Radiation therapy may also cause *fatigue* (feeling tired), which may persist for a few months after treatment stops.

Side Effects of Hormone Therapy

After orchiectomy *(castration),* about 90% of men have reduced or absent *libido* (sexual desire) and impotence. Over half of men have *hot flashes* (sudden rushes of body heat) after surgery, but these may go away with time.

Side effects of luteinizing hormone-releasing hormone (LHRH) agonists are the same as with an orchiectomy and include reduced or absent sexual desire, impotence, and hot flashes. Some men also have breast tenderness and growth of breast tissue (called gynecomastia). Breast growth usually occurs in men who take estrogen therapy. Using LHRH agonists over a long period of time can also cause osteoporosis (weakening of bones), fatigue, muscle wasting, and change in fat distribution. These side effects occur about as commonly as after orchiectomy.

A possible short-term side effect of LHRH agonists is what is known as *tumor flare*. When first given, these medicines can cause a temporary rise in testosterone levels, which can cause pain at tumor sites, especially if the cancer has already spread to bone. To prevent this, a short course of anti-androgens (at least 7 days) may be given when the LHRH agonist is first started.

Side effects of anti-androgens in patients already treated by orchiectomy or with LHRH agonists are usually not serious or common, but may include nausea, diarrhea, tiredness, liver disease, and the growth of breast tissue, especially with prolonged use.

Side Effects of Chemotherapy

The side effects of chemotherapy depend on the type of drugs, the amount taken, and the length of treatment. Temporary side effects might include nausea and vomiting, loss of appetite, hair loss, and mouth sores. Because chemo can damage the blood-producing cells of the bone marrow, patients may have low blood cell counts. This can result in an increased chance of infection (due to a shortage of white blood cells), excessive bleeding or bruising after minor cuts or injuries (due to a shortage of blood platelets), and fatigue (due to low red blood cell counts). Most side effects disappear once treatment is stopped. There are treatments for many of the short-term chemo side effects. For example, anti-nausea drugs can be given to prevent or reduce nausea and vomiting. Other drugs can be given to boost blood cell counts.

Considering Treatment Options

The stage of a cancer is one of the most important factors in selecting treatment options. The following section discusses treatment guidelines based on the AJCC (TNM) stage (see Prostate Cancer Stages on page 12).

Experts in prostate cancer treatment recommend that men consider treatment options in the context of their age and general health, goals for treatment, and views regarding side effects. Older men and those with other serious health problems often find it useful to think of prostate cancer as a chronic disease, one which will probably not kill them, but may cause symptoms they will wish to avoid. In this view, the goal is to relieve symptoms and avoid or reduce side effects of treatment. This view might lead some men to choose expectant management or hormone therapy. Radiation therapy is another good option for some patients. It provides a 5-year survival rate similar to radical prostatectomy.

On the other hand, men in their 40s, 50s, or 60s will also want to know about 10-year and 15-year survival rates. Postponing or relieving symptoms may not be their main goal. Instead, many younger, healthy men are more interested in a cure or at least in surviving beyond 10 to 20 years.

Personal feelings about side effects are another very important factor. Some men cannot imagine living with side effects such as incontinence or impotence. Other men are less concerned about these and more concerned about survival.

These difficult decisions are even harder for men who try to make them alone. Many men find that speaking with other men who have faced or are currently facing the same issues is helpful. The American Cancer Society's Man to Man® program and similar programs sponsored by cancer centers and other patient organizations provide a forum for men to meet and discuss these issues and concerns. For more information, call the ACS toll-free at 1-800-ACS-2345 or visit our Web site at www.cancer.org.

Other Things to Consider During and After Treatment

During and after treatment for your prostate cancer, you may be able to speed up your recovery and improve your quality of life by taking an active role. Learn about the benefits and disadvantages of each of your treatment options and ask questions of your cancer care team if there is anything you do not understand. Learn about and look out for side effects of treatment and report these promptly to members of your cancer care team so that they can take steps to reduce them.

Remember that your body is as unique as your personality and your fingerprints. Although understanding your cancer's stage and learning about your treatment options can help predict what health problems you may face, no one can say precisely how you will respond to cancer or its treatment.

You may have special strengths such as a history of excellent nutrition and physical activity, a strong family support system, or a deep faith, and these strengths may make a difference in how you respond to cancer and its treatment. There are also experienced professionals in mental health services, social work services, and pastoral services who may help you cope with your illness.

You can also help in your own recovery from cancer by making healthy lifestyle choices. If you use tobacco, stop now. Quitting will improve your overall health, and the full return of your sense of smell may help you enjoy a healthy diet during recovery. If you use alcohol, limit how much you drink. Have no more than 1 or 2 drinks per day. Good nutrition can help you get better after treatment. Eat a nutritious and balanced diet, with plenty of fruits, vegetables, and whole grain foods. If you are having eating problems, ask your cancer care team if you may benefit from talking with a dietitian.

If you are being treated for cancer, be aware of the battle that is going on in your body. Radiation therapy and chemotherapy add to the fatigue caused by the disease itself. To help combat the fatigue, plan your daily activities around when you feel your best and get plenty of sleep at night. Ask your cancer care team about a daily exercise program to help you feel better.

Concerns about sexuality are often very worrisome to a man with prostate cancer. Some treatments for prostate cancer can diminish sexual interest and/or response. Partner issues are also important because these changes affect the partner, as well as the patient. Partners are usually concerned about how to express their love physically and emotionally during and after treatment.

Suggestions that may help a man cope with these changes in his body include seeking the support of others, preferably before surgery; involving his partner as soon as possible after surgery; and openly communicating feelings, needs, and wants.

A cancer diagnosis and its treatment are major life challenges with an impact on you and everyone who cares for you. Before you reach the point of feeling overwhelmed, consider attending a meeting of a local support group. If you need other kinds of help, contact your hospital's social service department or the American Cancer Society.

About Clinical Trials

Studies of promising new or experimental treatments in patients are known as clinical trials. You may have heard about clinical trials being done for prostate cancer. Or maybe someone on your health care team has mentioned a clinical trial to you. Clinical trials are one way to get state-of-the art cancer care.

A clinical trial is only done when there is some reason to believe that the treatment being studied may be valuable to the patient. Treatments used in clinical trials are often found to have real benefits. Researchers conduct studies of new treatments to answer the following questions:

- Is the treatment helpful?
- What is the best way to give it?
- How does this new type of treatment work?
- Does it work better than other treatments already available?
- What side effects does the treatment cause?
- Are there more or fewer side effects than the standard treatment used now?
- Do the benefits outweigh the side effects?
- In which patients is the treatment most likely to be helpful?

Phases of Clinical Trials

There are 4 phases of clinical trials, which are numbered I, II, III, and IV.

Phase I clinical trials

The purpose of a phase I study is to find the best way to give a new treatment safely to patients. The cancer care team closely watches patients for any harmful side effects. Although doctors are hoping to help patients, the main purpose of such a phase I study is to test the safety of the treatment.

If a treatment is found to be reasonably safe in phase I studies, it can be tested in a phase II clinical trial.

Phase II clinical trials

These are designed to see if the treatment works. Patients are given the treatment as determined from phase I studies. They are closely watched for an effect on the cancer. The cancer care team also looks for side effects and benefits.

If a drug is found to be effective in phase II studies, it can be tested in a phase III clinical trial.

Phase III clinical trials

Phase III studies involve large numbers of patients. Some clinical trials may enroll thousands of patients. Often, these studies are randomized. This means that patients are randomly put in one of two (or more) groups. One group (called the control group) gets the standard, most accepted treatment. Another group (or more than one group) will get the new treatment being studied. All patients in phase III studies are closely watched. The study will be stopped early if the side effects of the new treatment are too severe or if one group has much better results than the others.

Phase III clinical trials are usually needed before the FDA will approve a treatment for use by the general public.

Phase IV clinical trials

Once a treatment has been approved by the FDA and is available for all patients, it is still studied in other clinical trials (sometimes referred to as phase IV studies). This way more can be learned about short-term and long-term side effects and safety as the treatment is used in larger numbers of patients with many types of diseases. Doctors can also learn more about how well the treatment works, and if it might be helpful when used in other ways (such as in combination with other treatments).

What It Will Be Like to Be in a Clinical Trial

If you are in a clinical trial, you will have a team of experts taking care of you and watching your progress very carefully. Depending on the phase of the clinical trial, you may receive more attention (such as having more doctor visits and lab tests) than you would if you were treated outside of a clinical trial. Clinical trials are especially designed to pay close attention to you.

However, there are some risks. No one involved in the study knows in advance whether the treatment will work or exactly what side effects will occur. That is what the study is designed to find out. While most side effects go away in time, some may be long-lasting or even life threatening. Keep in mind, though, that even standard treatments have side effects. Depending on many factors, you may decide to enter (enroll in) a clinical trial.

Deciding to Enter a Clinical Trial

If you would like to take part in a clinical trial, you should begin by asking your doctor if your clinic or hospital conducts clinical trials. There are requirements you must meet to take part in any clinical trial. But whether or not you enter (enroll in) a clinical trial is completely up to you.

Your doctors and nurses will explain the study to you in detail. They will go over the possible risks and benefits and give you a form to read and sign. The form says that you understand the clinical trial and want to take part in it. This process is known as giving your informed consent. Even after reading and signing the form and after the clinical trial begins, you are free to leave the study at any time, for any reason.

Taking part in a clinical trial does not keep you from getting any other medical care you may need.

To find out more about clinical trials, talk to your doctor, nurse, or other member of your cancer care team. Among the questions you should ask are:

- Is there a clinical trial that I could take part in?
- What is the purpose of the study?
- What kinds of tests and treatments does the study involve?
- What does this treatment do? Has it been used before?
- Will I know which treatment I receive?
- What is likely to happen in my case with, or without, this new treatment?
- What are my other choices and their pros and cons?
- How could the study affect my daily life?

- What side effects can I expect from the study? Can the side effects be controlled?
- Will I have to stay in the hospital? If so, how often and for how long?
- Will the study cost me anything? Will any of the treatment be free?
- If I am harmed as a result of the research, what treatment would I be entitled to?
- What type of long-term follow-up care is part of the study?
- Has the treatment been used to treat other types of cancers?

How Can I Find Out More About Clinical Trials That Might Be Right for Me?

The American Cancer Society offers a clinical trials matching service for patients, their family, and friends. You can reach this service at 1-800-303-5691 or on our Web site at http://clinicaltrials.cancer.org.

Based on the information you give about your cancer type, stage, and previous treatments, this service can put together a list of clinical trials that match your medical needs. The service will also ask where you live and whether you are willing to travel so that it can look for a treatment center that you can get to.

You can also get a list of current clinical trials by calling the National Cancer Institute's Cancer Information Service toll free at 1-800-4-CANCER (1-800-422-6237) or by visiting the NCI clinical trials Web site at www.cancer.gov/clinicaltrials.

For even more information on clinical trials, the American Cancer Society has a document called *Clinical Trials: What You Need to Know*. You can read this on the Web site, www.cancer.org, or have it sent to you by calling 1-800-ACS-2345.

NOTES

NOTES

Work-up (Evaluation) and Treatment Guidelines

Decision Trees

The Decision Trees (or flow charts) on the following pages represent different stages of prostate cancer. Each one shows you step-by-step how you and your doctor can arrive at the choices you need to make about your treatment.

Keep in mind, this information is not meant to be used without the expertise of your own doctor who is familiar with your situation, medical history, and personal preferences.

Participating in a clinical trial is an appropriate option for men at any stage of prostate cancer. Taking part in the study does not prevent you from getting other medical care you may need.

The NCCN guidelines are updated as new information becomes available. To ensure you have the most recent version, consult the Web sites of the ACS (www.cancer.org) or NCCN (www.nccn.org). You may also call the NCCN at 1-888-909-NCCN or the ACS at 1-800-ACS-2345 for the most recent information on these guidelines. If you have questions about your cancer or cancer treatment, please call the ACS any day at any time at 1-800-ACS-2345.

Initial Diagnosis

- DRE
- PSA blood test
- Biopsy
- Gleason score

Clinical Status

Life expectancy 5 years or less and no symptoms

Staging Work-up

No tests or treatments.

If symptoms expected within 5 years (T3, T4, or Gleason score of 8 or more), hormonal treatment or radiation may be given.

Clinical Status

Life expectancy of more than 5 years or symptoms present

Staging Work-up

Bone scan if T1, T2, and any of the following:

- PSA above 20, ***or***
- Gleason 8 or higher, ***or***
- There are symptoms, ***or***
- Tumor growing out of prostate gland (T3, T4).

CT or MRI of pelvis if:

- T1, T2, and lymph node spread is suspected, ***or***
- Tumor is growing out of prostate gland (T3, T4).

All others:
No additional testing

Prostate Cancer Work-up (Evaluation)

The NCCN guidelines for prostate cancer work-up begin by determining the size of the cancer by digital rectal exam (DRE), measuring the level of prostate-specific antigen (PSA), and determining the Gleason score in the biopsy specimen. The next issue is to estimate the patient's life expectancy and find out if the cancer is causing symptoms. Prostate cancer is often a chronic disease and men without symptoms may not benefit from immediate treatment, especially if they are in otherwise poor health. Talking about life expectancy, or how long a person is likely to live, is difficult, especially when it is your own life expectancy. But often other diseases are present that may determine how long a person might live. Remember that life expectancy is a guess. Usually the doctor's best guess is based on a

Prostate Cancer Work-up (Evaluation)

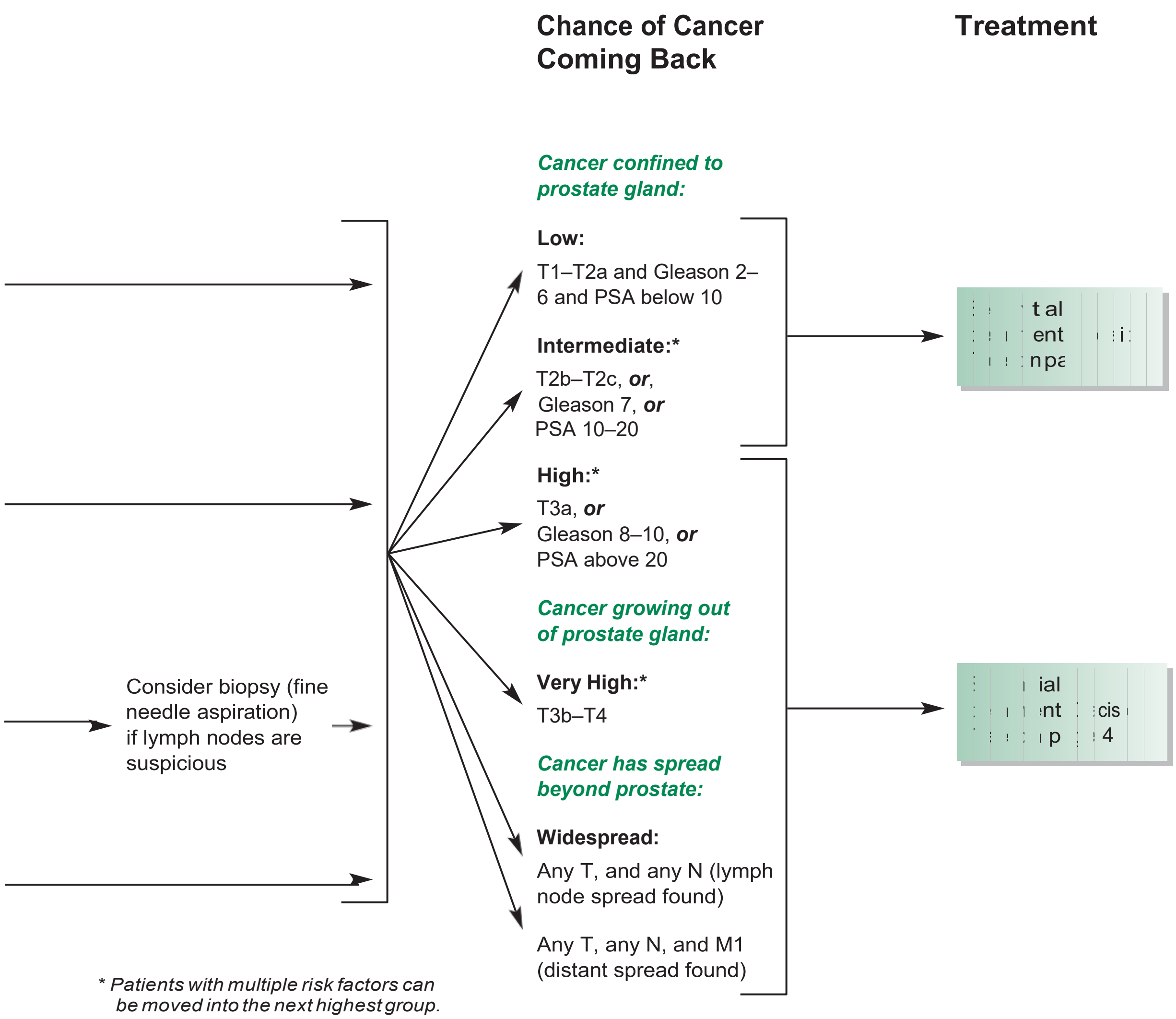

** Patients with multiple risk factors can be moved into the next highest group.*

©2007 by the National Comprehensive Cancer Network (NCCN) and the American Cancer Society (ACS). All rights reserved. The information herein may not be reproduced in any form for commercial purposes without the expressed written permission of the NCCN and the ACS. Single copies of each page may be reproduced for personal and non-commercial uses by the reader.

combination of estimated life expectancy by your age and what he or she knows about your health.

If the doctor thinks a man is likely to live 5 years or less and he has no symptoms, then there is no need for any tests or treatment. The exception to this is if the doctor thinks

the cancer will cause problems soon. This can happen if it looks like the cancer may block the urethra (the tube through which urine leaves the bladder) or spread rapidly. The warning sign for this is a cancer that is growing out of the prostate gland (T3 or T4) or a Gleason score of 8 or greater.

Additional testing is recommended for men expected to live 5 or more years or who have symptoms from the cancer. For example, if the tumor is T1 or T2, a bone scan is recommended if the PSA level is greater than 20 or if the Gleason score is greater than 8. A bone scan is also recommended if the man has any symptoms, or the cancer is growing outside the prostate (T3 or T4). A CT or MRI of the pelvis is recommended when the tumor is T1 or T2 and there is a 7% or greater chance of lymph node spread based on the Partin tables, or the tumor is growing outside the prostate (T3 or T4). A fine needle aspiration biopsy is considered if the CT or MRI scans show enlarged pelvic lymph nodes. The biopsy can confirm that the enlargement is due to spread of cancer (indicating N1 disease) rather than an infection or some other condition.

NOTES

Prostate Cancer Work-up (Evaluation) (continued)

All other stages need no additional testing as part of the work-up.

The next step is to estimate the risk of recurrence. This depends on the tumor size, the Gleason score, and the PSA level, as outlined in the Decision Tree. If the risk is low or intermediate, treatment is given as recommended in the Decision Tree "Initial Treatment for Prostate Cancer with Low to Intermediate Recurrence Risk" starting on page 36.

If the risk is high or very high, or the tumor has already spread to lymph nodes or distant sites, then treatment is outlined in the Decision Tree "Initial Treatment for Prostate Cancer with High to Very High Recurrence Risk or Spread to Lymph Nodes or Distant Sites" starting on page 40.

The intermediate and high risk categories include several risk factors. If a patient has more than one of these factors, then the risk level is shifted into the next higher group.

NOTES

Recurrence Risk	Life Expectancy	Initial Treatment
Cancer appears confined to prostate		
**Low:** T1–T2a, and Gleason 2–6, and PSA below 10	Less than 10 years	Observation (close follow-up – no immediate treatment) ***OR*** Radiation (3D-CRT or brachytherapy)
	10 years or more	Observation (close follow-up – no immediate treatment) ***OR*** Radiation (3D-CRT or brachytherapy) ***OR*** Radical prostatectomy with lymph node removal if risk of spread to lymph nodes is 7% or greater
**Intermediate*:** T2b–T2c, ***or*** Gleason 7, ***or*** PSA 10–20	Less than 10 years	Observation (close follow-up – no immediate treatment) ***OR*** Radiation (3D-CRT with or without brachytherapy) with lymph node removal if risk of spread to lymph nodes is 7% or greater ***OR*** Radical prostatectomy with lymph node removal if risk of spread to lymph nodes is 7% or greater
	10 years or more	Radical prostatectomy with lymph node removal if risk of spread to lymph nodes is 7% or greater ***OR*** Radiation (3D-CRT with or without brachytherapy) with lymph node removal if risk of spread to lymph nodes is 7% or greater

** Patients with multiple risk factors can be moved into the next highest group.*

Initial Treatment for Prostate Cancer With Low to Intermediate Recurrence Risk

Low risk: Low risk is defined as a tumor that either cannot be felt on digital rectal exam (DRE) or, if it is felt, it is in one half of one lobe or less; and the Gleason score is 2 to 6; and the prostate-specific antigen (PSA) level is below 10.

If the man is expected to survive less than 10 years, observation with close follow-up and

Adjuvant (Additional) Treatment

For men who had radical prostatectomy with cancer remaining after surgery:

- Rigorous observation (careful follow-up exams)

OR

- Radiation therapy

For men who had radical prostatectomy with spread of cancer to lymph nodes:

- Rigorous observation (careful follow-up exams)

OR

- Hormone therapy

See follow-up plan in the Decision Tree starting on page 44

©2007 by the National Comprehensive Cancer Network (NCCN) and the American Cancer Society (ACS). All rights reserved. The information herein may not be reproduced in any form for commercial purposes without the expressed written permission of the NCCN and the ACS. Single copies of each page may be reproduced for personal and non-commercial uses by the reader.

no immediate treatment is one option. Close follow-up involves:

- A DRE and PSA every 6-12 months depending on life expectancy
- A needle biopsy of the prostate gland may be repeated within 6 months if fewer than 10 cores were taken at the first biopsy or if the tumor appeared to be on the side opposite the positive biopsy site. The needle biopsy may be performed within 18 months if more than 10 cores were taken with the first biopsy.
- A repeat biopsy should be performed anytime it looks like the cancer is growing, based either on changes in DRE or PSA levels.

Another option would be radiation therapy – either a special type of external beam radiation called 3D-CRT, or brachytherapy.

If the life expectancy for the low-risk man is more than 10 years, the options are observation with close follow-up and no immediate treatment (close follow-up as described earlier), radiation therapy, or radical prostatectomy. Radiation therapy can be either 3D-CRT external beam radiation or brachytherapy. If prostatectomy is selected, the lymph nodes in the pelvis may be removed if the probability of tumor spread is at least 7%.

Intermediate risk: Intermediate risk is defined as a tumor that can be felt on DRE, and is in more than half of one lobe or in both lobes, or the Gleason score is 7, or the PSA is between 10 and 20.

If the life expectancy is less than 10 years, observation with close follow-up and no immediate treatment, radiation therapy with or without lymph node removal depending on risk of spread, or radical prostatectomy with or without lymph node removal are options.

NOTES

Initial Treatment for Prostate Cancer With Low to Intermediate Recurrence Risk (continued)

For patients expected to live more than 10 years, the NCCN advocates curative treatment because the cancer may eventually cause symptoms or shorten the life of these men. Radical prostatectomy or radiation are curative options. While brachytherapy alone is an option for low-risk patients, for intermediate-risk patients, 3D-CRT external beam radiation is recommended with or without additional brachytherapy. A pelvic lymph node dissection is also a consideration in all patients who have at least a 7% risk of tumor spread.

Additional treatment options are available for men if cancer remains after surgery (i.e., cancer is found at the edges [margins] of the specimen removed at prostatectomy). These men can either be carefully followed or may receive radiation therapy. If the cancer was found to have spread to lymph nodes at the time of surgery, radiation is not an option. Instead, close follow-up or hormone therapy are recommended.

On-going follow-up is discussed in the Decision Tree starting on page 44.

NOTES

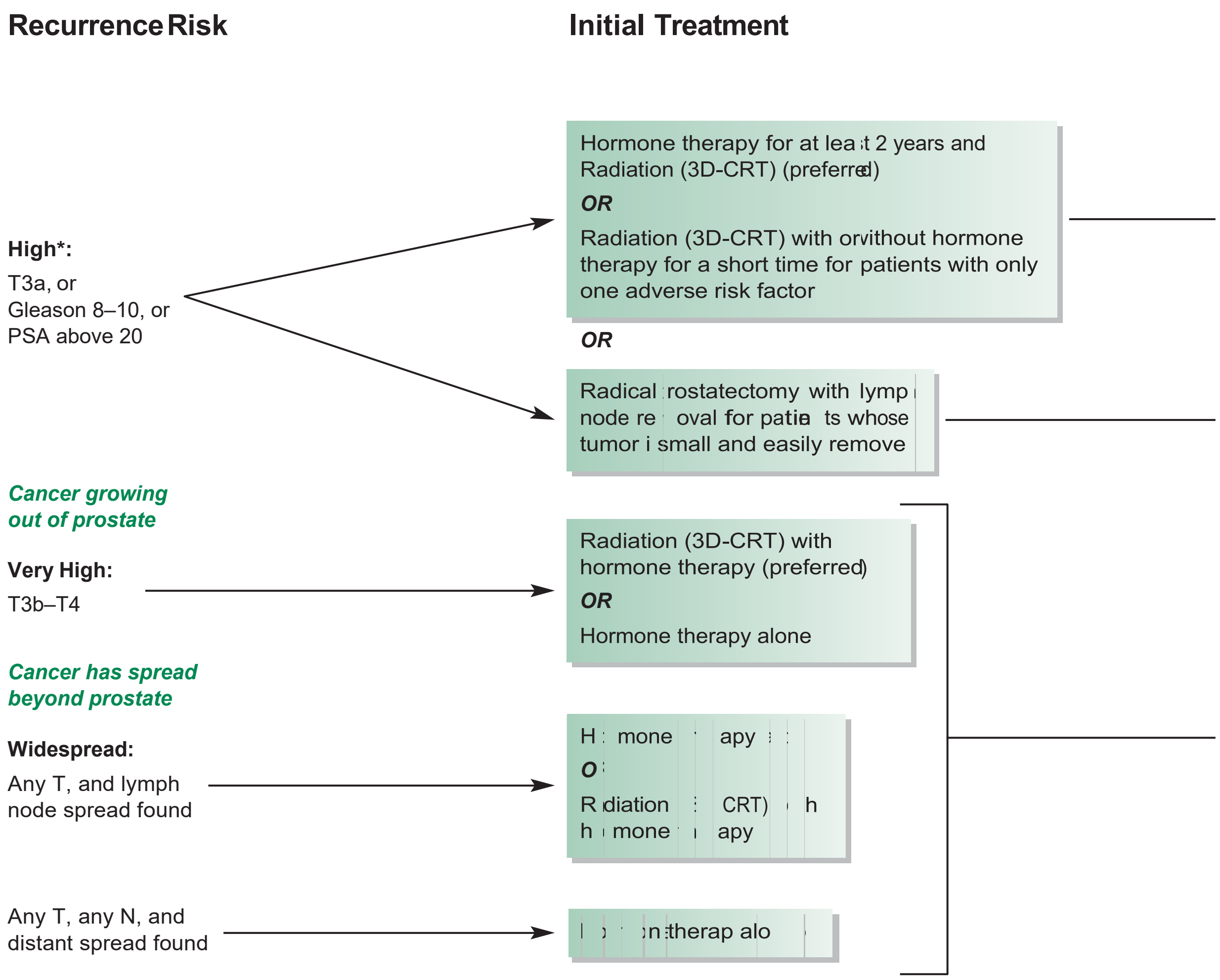

** Patients with multiple risk factors can be moved into the next highest group.*

Initial Treatment for Prostate Cancer With High to Very High Recurrence Risk or Spread to Lymph Nodes or Distant Sites

High risk: High risk is defined as a cancer growing outside the prostate but not into nearby tissues (T3a), or with a Gleason score of 8 to 10, or a PSA higher than 20. If a patient has more than one of these risk factors, the risk may be shifted to "very high risk" which is discussed below. For high risk, NCCN indicates that the preferred treatment is hormone therapy for at least 2 years along with 3D-CRT external beam radiation. For patients with a

Initial Treatment for Prostate Cancer With High to Very High Recurrence Risk or Spread to Lymph Nodes or Distant Sites

Adjuvant (Additional) Treatment

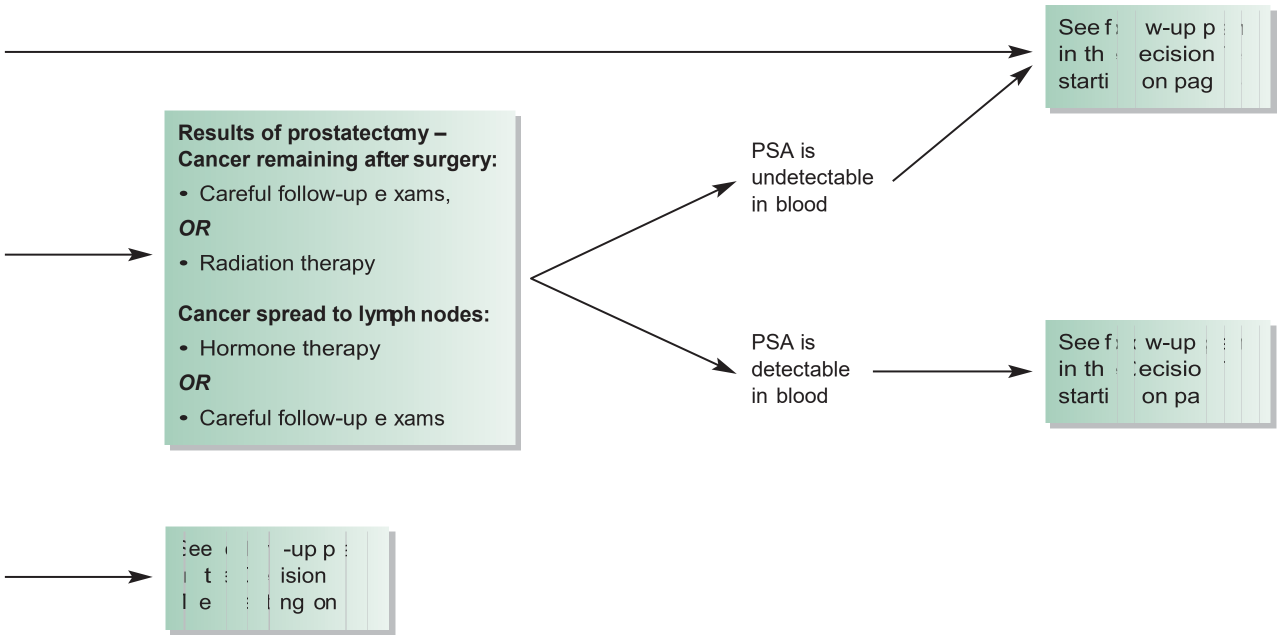

©2007 by the National Comprehensive Cancer Network (NCCN) and the American Cancer Society (ACS). All rights reserved. The information herein may not be reproduced in any form for commercial purposes without the expressed written permission of the NCCN and the ACS. Single copies of each page may be reproduced for personal and non-commercial uses by the reader.

single high risk factor, 3D-CRT external beam radiation with or without brief hormone therapy (given to shrink the cancer so radiation can be more effective) can be given. In patients whose tumor is small and easily removed, radical prostatectomy and lymph node removal is another option. After surgery, close follow-up or radiation therapy is recommended if the edges of the removed specimen contain cancer. If surgery reveals that the cancer has spread to the lymph nodes, either hormone therapy or close follow-up is recommended.

On-going follow-up or treatment is then determined by whether or not PSA is found in blood tests.

Very High risk: Very high risk is defined as cancer growing into nearby tissues (T3b to T4) or more than one of the risk factors described under high risk above. Surgery is not recommended. The preferred treatment is 3D-CRT external beam radiation combined with hormone therapy. Hormone therapy alone is another option.

On-going follow-up is discussed in the Decision Tree starting on page 44.

NOTES

Initial Treatment for Prostate Cancer With High to Very High Recurrence Risk or Spread to Lymph Nodes or Distant Sites (continued)

Widespread: If the cancer has spread to nearby lymph nodes, hormone therapy is recommended. 3D-CRT external beam radiation may be added to prevent local problems with the prostate cancer. If the cancer has spread to distant sites, only hormone therapy is recommended.

On-going follow-up is discussed in the Decision Tree starting on page 44.

NOTES

NCCN National Comprehensive Cancer Network

Treatment Guidelines for Patients

Initial Treatment **Follow-up**

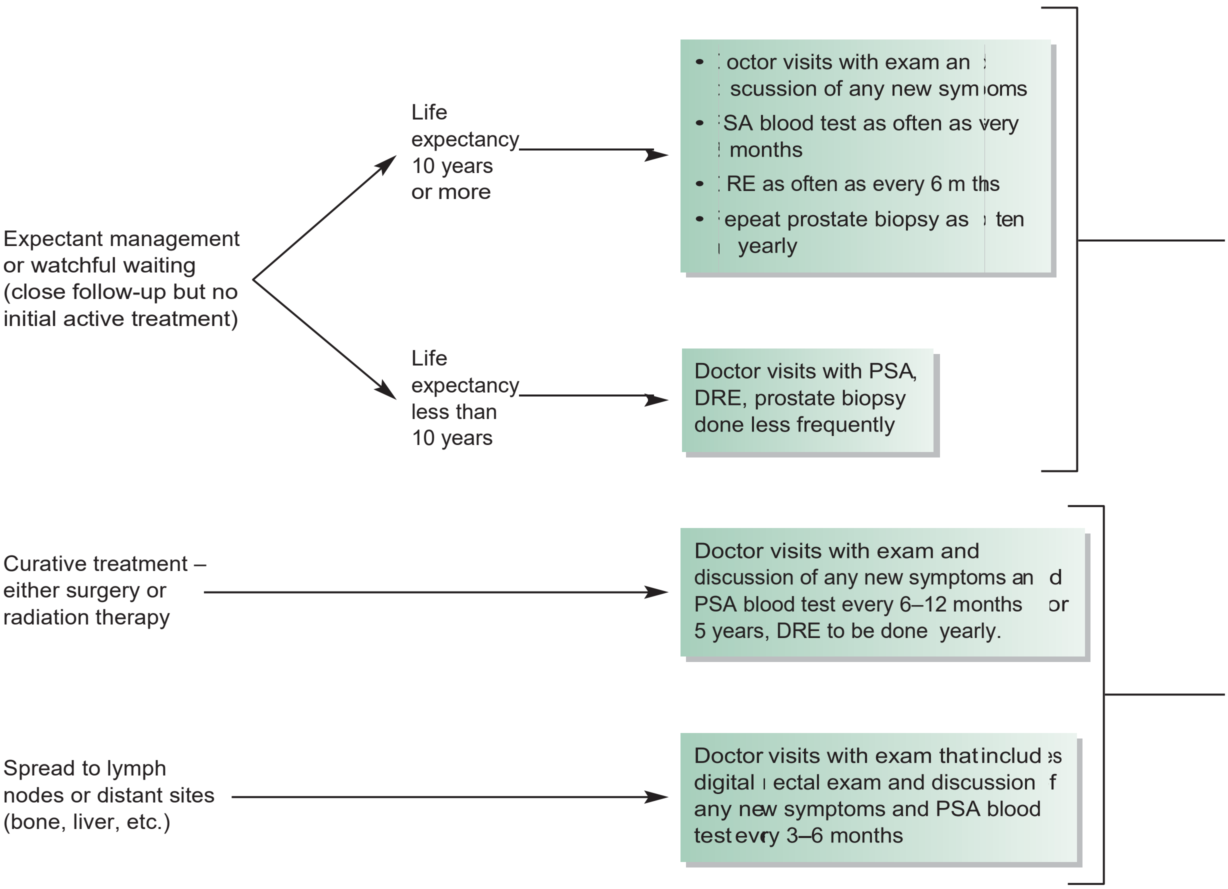

Follow-up *Surveillance* and Management of Recurrence

Follow-up of patients who are being managed with close observation depends on their life expectancy. If they are expected to live longer than 10 years, they should have regular doctor visits to discuss any new symptoms and have the following exams: prostate-specific antigen (PSA) blood tests as often as every 3 months, digital rectal exam (DRE) as often as every 6 months, and a repeat prostate biopsy may be done as often as once a year. If life expectancy is less than 10 years then routine doctor visits with PSA, DRE, and a prostate biopsy may be

Follow-up Surveillance and Management of Recurrence

Recurrence

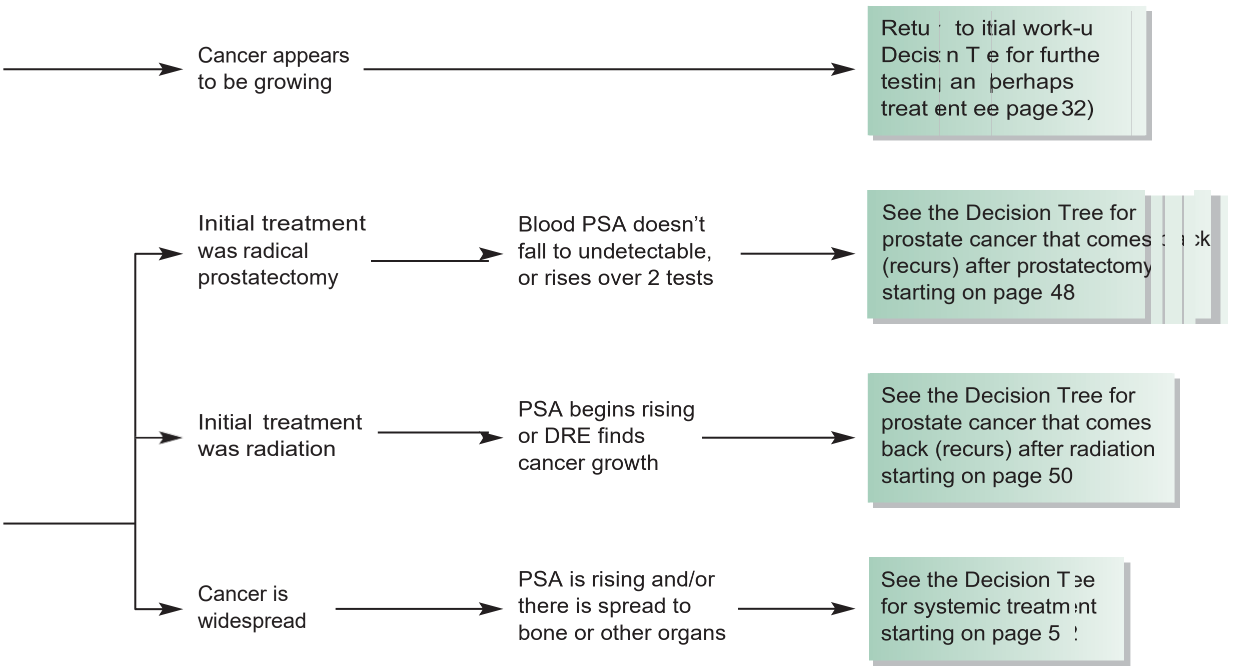

©2007 by the National Comprehensive Cancer Network (NCCN) and the American Cancer Society (ACS). All rights reserved. The information herein may not be reproduced in any form for commercial purposes without the expressed written permission of the NCCN and the ACS. Single copies of each page may be reproduced for personal and non-commercial uses by the reader.

performed less frequently. If there is evidence the cancer is growing, patients and physicians need to reevaluate the extent of the cancer as outlined in the first Decision Tree on page 32.

If the initial treatment was curative with either surgery or radiation, patients should see their doctor every 6 to 12 months for 5 years and then yearly. A PSA should be done at each visit and a DRE should be done every year.

If a radical prostatectomy was done, the PSA should fall to near 0. If this doesn't happen, or the PSA rises on 2 tests in a row, further

treatment may be needed as outlined in the Decision Tree "Work-up and Treatment for Prostate Cancer That Returns After Radical Prostatectomy" starting on page 48.

If the initial treatment was radiation, the PSA usually won't fall to 0, but will fall to a low level (usually less than or equal to 0.3 ng/mL). If the PSA increases by 2 ng/mL or cancer is found by DRE, more treatment will be needed as outlined in "Work-up and Treatment for Prostate Cancer that Returns After Radiation Therapy" starting on page 50.

NOTES

Follow-up Surveillance and Management of Recurrence (continued)

If the cancer had spread to lymph nodes or distant sites at the time of initial diagnosis, patients should be seen every 3 to 6 months for a discussion of symptoms, a physical examination, including DRE, and a PSA blood test.

If the PSA is rising or there is evidence of further spread, then treatment may be needed as described in the Decision Tree "Systemic Treatment for Widespread Prostate Cancer" which starts on page 52.

NOTES

Abnormal Follow-up Results After Radical Prostatectomy

PSA levels do not fall to zero

PSA levels increasing on 2 or more tests

Work-up (Evaluation)

Consider:
- Bone scan
- Biopsy of the area from which the prostate was removed
- CT or MRI scan of the pelvis
- ProstaScint® scan

Likely to Benefit from Radiation

Patient has a PSA of 2 or less before radiation and fits one of the following categories:
- Gleason score 7 or less, tumor found at the edge of the prostatectomy
- Gleason score 7 or less, no tumor after prostatectomy, but PSA level is rising quickly
- Gleason score 8–10, tumor found at the edge of prostatectomy, but PSA level is rising quickly

Less Likely to Benefit from Radiation

Tumor spread to seminal vesicle or lymph nodes, or does not fall into one of the categories above

Cancer spread beyond prostate and adjacent lymph nodes

Work-up and Treatment for Prostate Cancer That Returns After Radical Prostatectomy

After a prostatectomy, further tests are recommended if PSA doesn't fall to near 0, or if the PSA is detectable and then rises on 2 or more tests. These PSA results suggest that the cancer has returned. The tests that are recommended are designed to find the site of recurrence. A bone scan determines whether the cancer has spread to the bone; a biopsy can detect recurrence in the area of the prostate; and a CT, MRI, or ProstaScint® scan can look for spread to lymph nodes or other organs, such as the liver.

If the cancer has recurred only in the area of the prostate, external beam radiation therapy is an option. As noted in the Decision Tree, there are some patients that are more likely to benefit from radiation than others based on the Gleason score, the PSA level, and how fast the PSA is rising. External beam radiation

Work-up and Treatment for Prostate Cancer That Returns After Radical Prostatectomy

Treatment

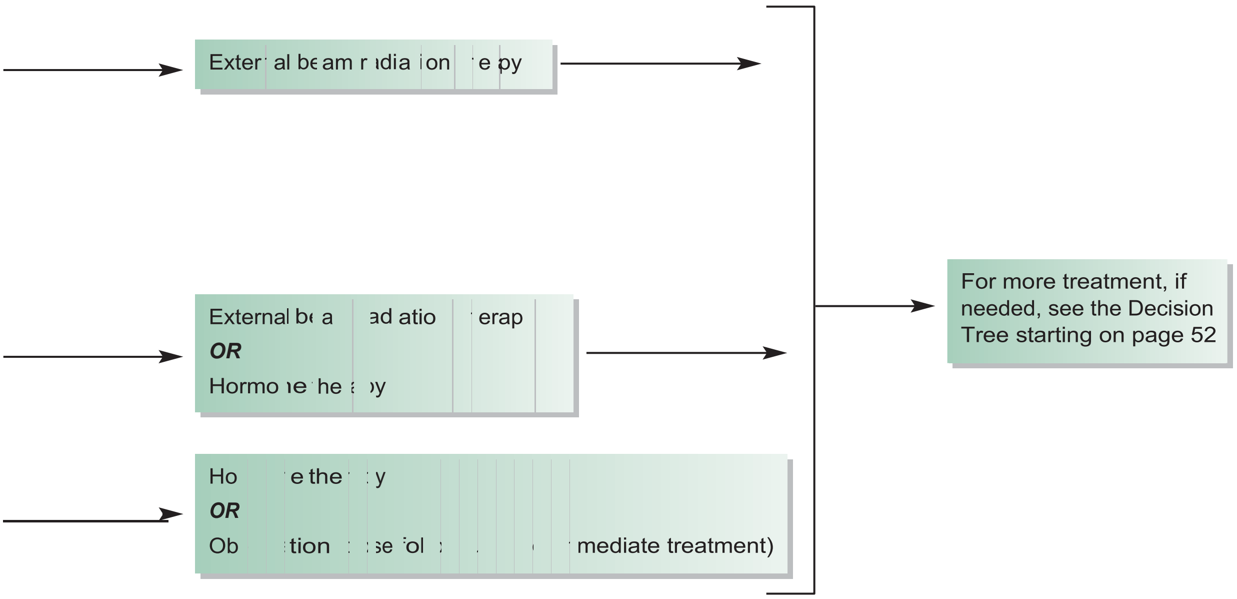

©2007 by the National Comprehensive Cancer Network (NCCN) and the American Cancer Society (ACS). All rights reserved. The information herein may not be reproduced in any form for commercial purposes without the expressed written permission of the NCCN and the ACS. Single copies of each page may be reproduced for personal and non-commercial uses by the reader.

therapy is offered to patients most likely to benefit, while hormone therapy is an alternative option for those less likely to benefit from radiation, including those with a tumor involving the lymph nodes or the seminal vesicles. Patients with metastasis beyond the prostate and lymph nodes are offered either hormone therapy, or may be followed with no immediate treatment. The NCCN recommends participation in clinical trials also be encouraged.

If more treatment is needed, then systemic treatment should be given as outlined in the Decision Tree "Systemic Treatment for Widespread Prostate Cancer" which starts on page 52.

Abnormal Follow-up Results | **Work-up (Evaluation)**

After radiation therapy:

- PSA levels increase by 2 ng/ml

OR

- Positive DRE

→ Possible candidate for local treatment such as radical prostatectomy surgery because:

- Cancer was early stage (T1–T2) with no evidence of lymph node spread
- Life expectancy greater than 10 years
- PSA now less than 10

→

- Biopsy of prostate area
- Bone scan
- Consider CT or MRI scan of the abdomen and pelvis
- Consider ProstaScint® scan

→ Not a candidate for local treatment such as radical prostatectomy surgery

Work-up and Treatment for Prostate Cancer That Returns After Radiation Therapy

After radiation therapy, if the prostate-specific antigen (PSA) rises by 2 points or more or there is a positive result on a digital rectal exam (DRE), it is very likely the cancer is coming back. The first step is to determine whether or not the patient is a candidate for a prostatectomy or other local therapies, such as *cryosurgery*. Potential candidates include men who had a localized cancer (T1–T2) with no evidence of lymph node spread or distant spread, who are expected to live 10 or more years, and have PSA levels less than 10. First the prostate area is biopsied, along with a bone scan to rule out spread to the bones. A CT scan or MRI of the abdomen and pelvis and ProstaScint® scan may also be done. If the biopsy shows cancer and there is no sign that the cancer has spread, radical prostatectomy or other local therapies, preferrably in a clinical trial, are options. If the cancer has spread to lymph nodes or other sites, then

Work-up and Treatment for Prostate Cancer That Returns After Radiation Therapy

Treatment

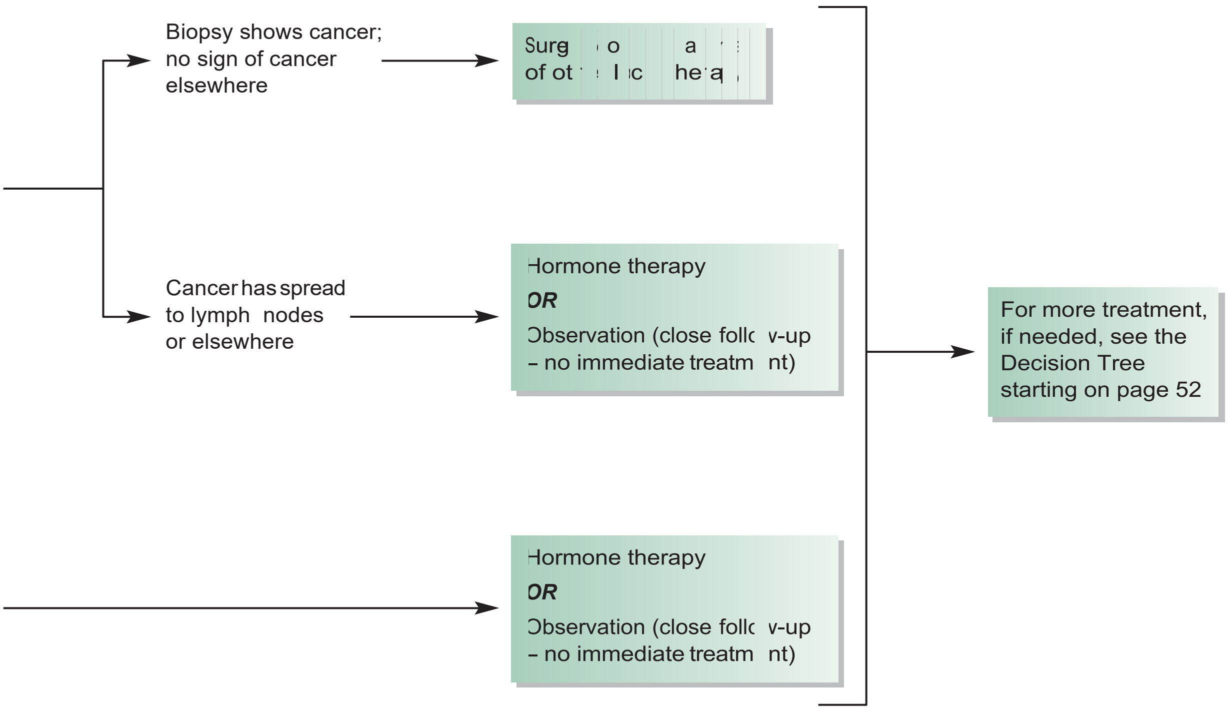

©2007 by the National Comprehensive Cancer Network (NCCN) and the American Cancer Society (ACS). All rights reserved. The information herein may not be reproduced in any form for commercial purposes without the expressed written permission of the NCCN and the ACS. Single copies of each page may be reproduced for personal and non-commercial uses by the reader.

hormone therapy, close observation without immediate treatment, or participation in a clinical trial are appropriate options.

If the patient is not a candidate for radical prostatectomy because the original tumor was larger than T1 or T2; or the PSA level is above 10; or for other reasons, such as ill health, expected short survival, or personal preference, then hormone therapy, close observation without immediate treatment, or participation in a clinical trial are appropriate options.

If more treatment is needed, it should be given according to the "Systemic Treatment for Widespread Prostate Cancer" Decision Tree which starts on page 52.

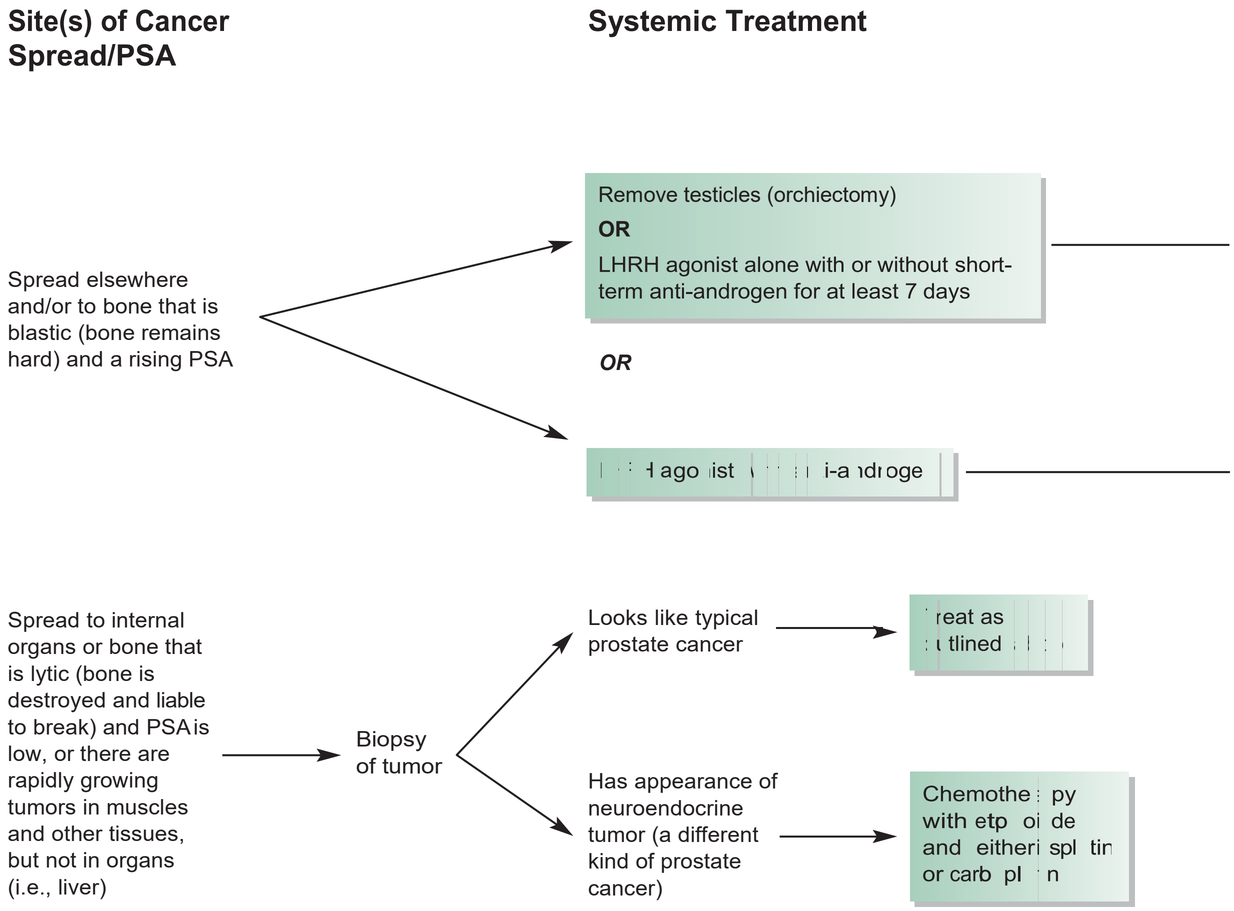

Systemic Treatment for Widespread Prostate Cancer

If the cancer has spread to bone and the x-rays of bone show a typical appearance of denser (harder) bone, or there is spread to other organs, and the PSA is rising, the first treatment should be hormone therapy. One option is orchiectomy. Another option is to use a luteinizing hormone-releasing hormone (LHRH) agonist. This may be used with at least 7 days of an anti-androgen to prevent tumor flare. If there is relapse after orchiectomy or LHRH agonist therapy, then anti-androgens; other hormone therapy, such as ketoconazole with or without cortisone-like drugs; or estrogen could be used.

Systemic Treatment for Widespread Prostate Cancer

Further Treatment If Cancer Returns

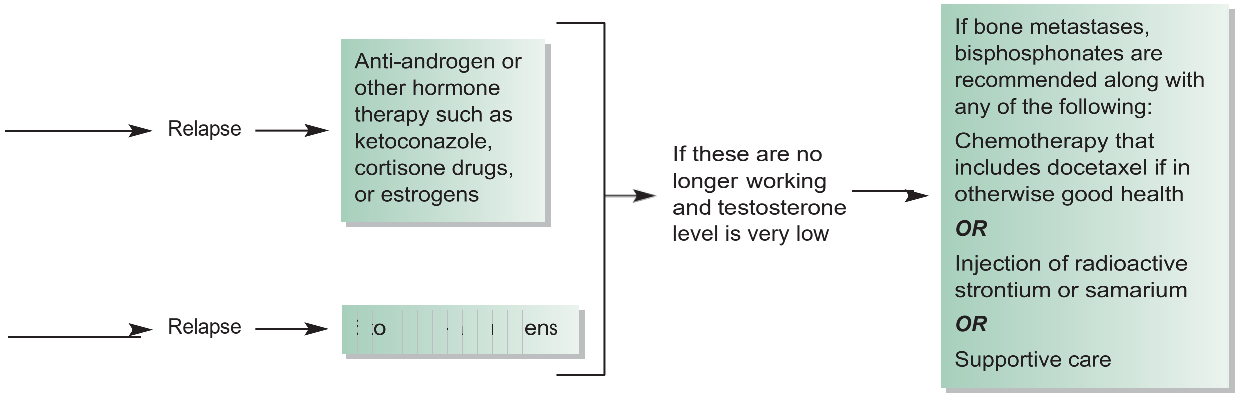

©2007 by the National Comprehensive Cancer Network (NCCN) and the American Cancer Society (ACS). All rights reserved. The information herein may not be reproduced in any form for commercial purposes without the expressed written permission of the NCCN and the ACS. Single copies of each page may be reproduced for personal and non-commercial uses by the reader.

If an anti-androgen has been used along with the LHRH agonist when the relapse occurs, the anti-androgen must be stopped. This is because sometimes anti-androgens begin stimulating the cancer to grow.

If these treatments stop working and the testosterone level remains low, there are other treatment options. Chemotherapy that includes docetaxel along with other agents is an option for men in fairly good health. If the cancer is confined to bones, the radioactive drugs samarium or strontium can be helpful. Bisphosphonates should be given to men with bone metastases. These drugs can help prevent bone fracture and may prevent further bone spread.

Sometimes the cancer causes bones to soften (lytic metastases). A biopsy of the site should be done, along with a biopsy of any isolated spread to an organ or rapidly growing tumors in soft tissues like muscle. If the biopsy shows typical prostate cancer, the treatment outlined above should be given. If the biopsy shows small cell or neuroendocrine cancer then chemotherapy with etoposide and either cisplatin or carboplatin is recommended. Hormone therapy isn't usually successful and is not recommended in this situation.

For men who decide they do not want further anti-cancer treatment, *supportive care* with only treatment of symptoms is an option. Supportive care includes treatments that don't affect the cancer's growth but are given to relieve symptoms, for example pain medications. It is important to remember, especially in this situation, that the goal of prostate cancer treatment is to make the patient feel as well as possible. Removing or destroying all of the cancer cells is one way to accomplish that goal. But even when that is no longer

NOTES

possible, patients still have other options for relieving symptoms. There is no reason to endure pain or other symptoms when supportive care treatments are available. Some patients assume that symptoms of advanced prostate cancer cannot be effectively treated. This is not the case. Discuss any symptoms you may have with your cancer care team. If you do not do this you may miss out on opportunities to maintain your best quality of life for as long as possible.

NOTES

Glossary

Adjuvant treatment

Treatment used in addition to the main treatment. It usually refers to hormone therapy, chemotherapy, or radiation added after surgery to increase the chances of destroying all of the cancer or keeping it in check.

Alkaline phosphatase

An enzyme made by cells in the bones and liver. Levels of alkaline phosphatase in the blood often go up in men whose prostate cancer has spread to the bones or liver.

Androgen

Any male sex hormone. The major androgen is testosterone. See testosterone.

Androgen deprivation therapy

The use of drugs to block the actions of androgens (male hormones). See hormone therapy.

Androgen-independent cancers

These are prostate cancers that do not respond to hormone therapy. Often prostate cancers respond to hormone therapy for a few years before becoming androgen-independent. Less often, prostate cancers may be androgen-independent when they are diagnosed. Prostate cancer that starts off being androgen-independent may be a different kind of prostate cancer called small cell or neuroendocrine. See neuroendocrine prostate cancer and small cell prostate carcinoma.

Anti-androgens

Drugs that block the body's ability to use androgens. Several drugs of this type are currently available. Anti-androgens are usually combined with orchiectomy or LHRH agonists. See androgen, orchiectomy, and LHRH agonists.

Benign prostatic enlargement

This is non-cancerous enlargement of the prostate that may cause problems with urination, such as trouble starting and stopping the flow. These symptoms are called "lower urinary tract symptoms" or LUT's. They are usually due to benign prostate enlargement (BPE). BPE is usually caused by benign prostatic hyperplasia (BPH) that is a histological diagnosis (a pathologist makes this diagnosis by looking at the removed prostate tissue).

Biopsy

The removal of a sample of tissue to see if cancer cells are present. There are several kinds of biopsies. See fine needle aspiration and core needle biopsy.

Bisphosphonates

A group of medicines that can slow the damage caused by cancer spread within bones, reduce the risk of bone fractures, and may relieve pain caused by bone metastases. The bisphosphonates most commonly used are pamidronate and zoledronic acid.

Blastic
Bone metastases that make the bone appear denser and harder. This term is taken from osteoblastic (osteoblasts are cells that make bone). Compare to lytic.

Brachytherapy
Internal radiation treatment given by placing radioactive material directly into the tumor or very close to it. May also be called interstitial radiation therapy or seed implantation. Compare to external beam radiation.

Capsule
The rim of tissue surrounding the prostate or other organs.

Castration
Surgery to remove the testicles; the medical term is orchiectomy.

Catheter
A thin, flexible tube through which fluids enter or leave the body; in prostate cancer most commonly a tube threaded through the penis into the bladder to drain urine.

Chemotherapy
Treatment with drugs to destroy cancer cells. Chemotherapy is often used with surgery or radiation to treat cancer when it has spread, when it has come back (recurred), or when there is a strong chance that it could recur.

Clinical stage
Describes the extent of cancer present based on results of diagnostic tests and the physical examination. Compare to pathologic stage.

Clinical trial
A study of promising new or experimental treatments in patients

Combination hormone therapy
Complete blockage of androgen action that may include orchiectomy (castration) or luteinizing hormone-releasing hormone agonists, plus the use of anti-androgens. Also called combined androgen blockade, total hormonal ablation, total androgen blockade, or total androgen ablation.

CT or CAT scan (computed tomography)
An imaging test in which many x-ray images are taken from different angles of a part of the body. These images are combined by a computer to produce cross-sectional pictures of internal organs.

Core needle biopsy
A procedure in which the doctor uses a narrow needle to remove a cylinder of tissue, usually about ½-inch long and $\frac{1}{16}$-inch across, which is sent to the laboratory and examined under a microscope to see if cancer is present.

Cryosurgery
The use of extreme cold to freeze and destroy cancer cells. Also called cryoablation.

Digital rectal examination
Also called DRE, it is an exam in which the doctor inserts a gloved finger into the rectum to feel for anything not normal. Some tumors of the rectum and prostate gland can be felt during a DRE.

Early detection
Finding the disease at an early stage, before it has grown large or spread to other sites. Many forms of cancer can reach an advanced stage without causing symptoms. Mammography can help to find breast cancer early, and the PSA blood test can help to find early prostate cancer.

Expectant management
See watchful waiting.

External beam radiation
A form of treatment in which radiation is focused from a source outside the body on the area affected by the cancer. It is much like getting a diagnostic x-ray, but for a longer time. Compare to brachytherapy.

Fatigue
This is a common symptom during cancer treatment, described as a bone-weary exhaustion that doesn't get better with rest. For some, this can last for some time after treatment.

Fine needle aspiration
In this procedure, a thin needle is used to draw up (aspirate) samples for examination under a microscope. Fine needle aspiration is sometimes used to determine if prostate cancer has spread to lymph nodes inside the pelvis.

Gland
A group of cells that produce and release substances used nearby or in another part of the body. The prostate is a gland.

Gleason score
A method of classifying prostate cancer cells on a scale of 2 to 10. The higher the Gleason score (also called Gleason sum), the faster the cancer is likely to grow and the more likely it is to spread beyond the prostate. See Gleason system.

Gleason system
The most often used prostate cancer grading system. This system assigns a Gleason grade ranging from 1 through 5 based on how much the arrangement of the cancer cells look the way normal prostate cells are arranged in the prostate gland. Because prostate cancers often have areas with different grades, a grade is assigned to the 2 areas that make up most of the cancer. These 2 grades are added to give a Gleason score between 2 and 10. See Gleason score.

Hormone
A chemical substance released into the body by the endocrine glands such as the thyroid, adrenals, testicles, or ovaries. The substance travels through the bloodstream and sets in motion various body functions. Testosterone and estrogen are examples of male and female hormones.

Hormone therapy
Treatment with hormones, drugs that interfere with hormone production or hormone action, or the surgical removal of hormone-producing glands. Hormone therapy may kill cancer cells or slow their growth. In prostate cancer treatment, hormone therapy is sometimes called androgen deprivation. See androgen deprivation therapy.

Hot flash
Sudden rush of body heat causing reddening and sweating; a common side effect of some types of hormone therapy.

Impotence
Also called erectile dysfunction (ED), it is the inability to get an erection of the penis.

Incontinence, urinary
The inability to control the urine stream, resulting in leakage or dribbling of urine. Incontinence is divided into 3 types: stress incontinence, overflow incontinence, and urge incontinence. See also urinary urgency.

Laparoscope
A long, slender tube inserted into the abdomen through a very small incision. The laparoscope allows the surgeon to view organs and lymph nodes within the body. The lymph nodes, or even the prostate gland itself, can be removed using special surgical instruments operated through the laparoscope.

Libido
Sex drive.

Luteinizing hormone
Pituitary hormone that stimulates the testicles to produce testosterone. See luteinizing hormone-releasing hormone.

Luteinizing hormone-releasing hormone A hormone produced by the hypothalamus, a tiny gland in the brain, which indirectly affects testosterone levels. See luteinizing hormone.

Luteinizing hormone-releasing hormone agonists
These are synthetically made hormones, chemically similar to LHRH. They block the production of the male hormone testosterone and are sometimes used to treat prostate cancer. Also called LHRH analogs.

Lymph nodes
Small, bean-shaped collections of immune system cells that help fight infections and also have a role in fighting cancer. Also called lymph glands.

Lytic
Bone metastases that appear to dissolve the bone. Compare to blastic.

Metastasis
The spread of cancer cells to distant areas of the body by way of the lymph system or bloodstream.

Neuroendocrine prostate cancer
An uncommon cancer that arises from a specialized neuroendocrine cell in the prostate gland. Some of these cancers are called small cell cancers. A similar cancer develops in the lungs and in other organs in the body. See small cell prostate carcinoma.

Observation
See watchful waiting.

Orchiectomy
Surgery to remove the testicles; castration.

Partin tables
Tables based on a large group of previous cases, used to predict the likelihood that prostate cancer has spread to the lymph nodes or other organs. The tables take into account the clinical stage and Gleason score of a man's cancer, as well as his PSA level.

Pathologic stage
Describes the extent of cancer present based on surgical removal and examination of tissue. Compare to clinical stage.

Pathologist
A doctor who specializes in diagnosis and classification of diseases by laboratory tests such as examination of tissue and cells under a microscope. The pathologist determines whether a tumor is benign or cancerous, and, if cancerous, the exact cell type and grade.

Prognosis
A prediction of the course of disease; the outlook for the chances of survival.

Prostate-specific antigen
A protein made in large quantities by the prostate gland but also made by many other tissues including small intestines, breasts, and salivary and parotid glands. Levels of prostate-specific antigen (PSA) in the blood often go up in men with prostate cancer but may increase because of some noncancerous conditions too. The PSA test is used to help find prostate cancer, as well as to monitor the results of treatment.

Radical prostatectomy
Surgery to remove the entire prostate gland, the seminal vesicles, and nearby tissue.

Seminal vesicles
Two small sacs next to the prostate that store semen.

Small cell prostate carcinoma
An uncommon form of prostate cancer. See also neuroendocrine prostate cancer.

Stage
Describes how much cancer is present. Determining the stage is essential for choosing the best treatment. The stage is often described using the TNM classification system, where T stands for tumor (size and how far it has spread to nearby organs), N stands for spread to lymph nodes, and M is for metastasis. Letters or numbers after the T, N, and M provide more details about each of these factors. See clinical stage and pathologic stage.

Supportive care
Measures taken to relieve symptoms and improve quality of life, but not expected to destroy the cancer. Pain medicine is an example of supportive care.

Surveillance
See watchful waiting.

Testicles
The male reproductive glands found in the scrotum. The testes (or testicles) produce sperm and the male hormone testosterone.

Testosterone
The main male hormone, made primarily in the testes. It stimulates blood flow, growth of certain tissues, and the secondary sexual characteristics. In men with prostate cancer, it can also encourage growth of the tumor. See androgen.

Three-dimensional conformal radiation therapy
This treatment uses sophisticated computers to map very precisely the location of the cancer within the prostate. The patient may be fitted with a plastic mold resembling a body cast to keep him still so that the radiation can be more accurately aimed. Radiation beams are then aimed from several directions.

Transurethral resection of the prostate
Also called TURP, this operation removes part of the prostate gland that surrounds the urethra (the tube through which urine exits the bladder). TURP can be used to relieve symptoms caused by a tumor before other treatments begin, but it is not expected to cure disease or remove all of the cancer. It is used even more often to relieve symptoms of noncancerous prostate enlargement.

Tumor flare
Temporary growth of a prostate tumor, sometimes seen in men who are treated with a luteinizing hormone-releasing hormone agonist alone. The growth is due to the brief rise in testosterone levels after starting treatment. It can cause pain, especially if cancer has already spread to the bones. It often takes a few weeks before testosterone levels begin to fall. Tumor flare can be prevented by taking an anti-androgen for the first few weeks of hormone therapy.

Urinary urgency
Feeling that you need to urinate right away.

Urologist
A doctor who specializes in treating problems of the urinary tract in men and women, and of the genital area in men. Urologists often perform surgery.

Watchful waiting
Instead of active treatment for prostate cancer, the doctor may suggest close monitoring. This may be a reasonable choice for older men with small tumors that might grow very slowly. If the situation changes, active treatment can be started. Also called expectant management, watchful expectancy, observation, or surveillance.

For a more comprehensive glossary, you may access the American Cancer Society Web site at www.cancer.org.

NOTES

NOTES

Current Cancer Treatment Guidelines for Patients

Advanced Cancer and Palliative Care Treatment Guidelines for Patients (English and Spanish)

Bladder Cancer Treatment Guidelines for Patients (English and Spanish)

Breast Cancer Treatment Guidelines for Patients (English and Spanish)

Cancer Pain Treatment Guidelines for Patients (English and Spanish)

Cancer-Related Fatigue and Anemia Treatment Guidelines for Patients (English and Spanish)

Colon and Rectal Cancer Treatment Guidelines for Patients (English and Spanish)

Distress Treatment Guidelines for Patients (English and Spanish)

Fever and Neutropenia Treatment Guidelines for Patients with Cancer (English and Spanish)

Lung Cancer Treatment Guidelines for Patients (English and Spanish)

Melanoma Cancer Treatment Guidelines for Patients (English and Spanish)

Nausea and Vomiting Treatment Guidelines for Patients with Cancer (English and Spanish)

Non-Hodgkin's Lymphoma Treatment Guidelines for Patients (English and Spanish)

Ovarian Cancer Treatment Guidelines for Patients (English and Spanish)

Prostate Cancer Treatment Guidelines for Patients (English and Spanish)

The *Prostate Cancer Treatment Guidelines for Patients* were developed by a diverse group of experts and were based on the NCCN clinical practice guidelines. These patient guidelines were translated, reviewed, and published with help from the following individuals:

Terri Ades, MS, APRN-BC, AOCN
American Cancer Society

Kimberly A. Stump-Sutliff, MS, RN, AOCNS
American Cancer Society

Joan McClure, MS
National Comprehensive Cancer Network

Dorothy A. Shead, MS
National Comprehensive Cancer Network

Elizabeth Brown, MD
National Comprehensive Cancer Network

James Mohler, MD
Roswell Park Cancer Institute

The *NCCN Prostate Cancer Clinical Practice Guidelines* and *Prostate Cancer Early Detection Clinical Practice Guidelines* were developed by the following NCCN Panel Members:

Richard J. Babaian, MD
The University of Texas M.D. Anderson Cancer Center

Robert R. Bahnson, MD
Arthur G. James Cancer Hospital & Richard J. Solove Research Institute at The Ohio State University

Barry Boston, MD
St. Jude Children's Research Hospital/University of Tennessee Cancer Institute

Anthony D'Amico, MD, PhD
Dana Farber/Brigham and Women's Cancer Center | Massachusetts General Hospital Cancer Center

James A Eastham, MD
Memorial Sloan-Kettering Cancer Center

Ralph J. Hauke, MD
UNMC Eppley Cancer Center at The Nebraska Medical Center

Robert P. Huben, MD
Roswell Park Cancer Institute

Philip Kantoff, MD
Dana-Farber/ Brigham and Women's Cancer Center | Massachusetts General Hospital Cancer Center

Mark Kawachi, MD
City of Hope

Michael Kuettel, MD, PhD, MB
Roswell Park Cancer Institute

Paul H. Lange, MD
Fred Hutchinson Cancer Research Center/Seattle Cancer Care Alliance

Chris Logothetis, MD
The University of Texas M.D. Anderson Cancer Center

Gary MacVicar, MD
Robert H. Laurie Comprehensive Cancer Center at Northwestern University

James Mohler, MD
Roswell Park Cancer Institute

Alan Pollack, MD, PhD
Fox Chase Cancer Center

Julio M. Pow-Sang, MD
H. Lee Moffitt Cancer Center and Research Institute at the University of South Florida

Mack Roach, III, MD
UCSF Comprehensive Cancer Center

Howard Sandler, MD
University of Michigan Comprehensive Cancer Center

Dennis Shrieve, MD
Huntsman Cancer Institute at the University of Utah

Sandra Srinivas, MD
Stanford Comprehensive Cancer Center

Przemyslaw Twardowski, MD
City of Hope

Donald A. Urban, MD
University of Alabama at Birmingham Comprehensive Cancer Center

Patrick C. Walsh, MD
The Sidney Kimmell Comprehensive Cancer Center at Johns Hopkins

©2007, American Cancer Society, Inc.
No.950403

American Cancer Society®

1.800.ACS.2345
www.cancer.org

NCCN National Comprehensive Cancer Network

1.888.909.NCCN
www.nccn.org

Appendix B: Content Items Present or Absent in the High Literacy and Plain Language DAs.

	Standard DA	Patient Centered DA
Individual Patient Characteristics		
**Effects of age upon**		
Treatment	1	1
**Race/Ethnicity**		
Treatment	0	0
Outcome	0	0
Clinical Condition		
**Physiology**		
What is the prostate?	1	1
Where is the prostate?	1	1
What is its function?	1	1
Picture/Image	1	1
**Natural History of Disease**		
Description of cancer	1	1
Death	1	1
Distinguish benign/metastatic	0	1
**Pathology - Stages**		
Staging - Definition	1	1
Gleason- Definition	1	1
Watchful Waiting		
**Procedure**		
Description of procedure	1	1
**Patient Experience**	1	1
Patient testimonial	0	1
**Presentation of risk**		
Pros and Cons	1	1
Pros and Cons Quantitative	0	1
Framing	0	1
Radical Prostatectomy		
**Procedure**		
Description of procedure	1	1
Mentions perineal approach	1	1

Mentions retropubic approach	1	1
Mentions nerve sparing operation	1	1
**Side Effects**		
Bowel problems	0	0
Bowel: Distinguish temporary/permanent	0	0
Bladder incontinence	1	1
Bladder: Distinguish temporary/permanent	1	1
Impotence	1	1
Impotence: Distinguish temporary/permanent	1	1
Death	0	1
**Patient Experience**		
Hospitalization necessary	1	1
Cathaterization necessary	1	1
Patient testimonial	0	1
**Presentation of risk**		
Pros and Cons	1	1
Pros and Cons Quantitative	1	1
Framing	0	1
Radiation		
**Procedure**		
Description of procedure	1	1
Distinguish internal/external	1	1
**Side Effects**		
Bowel problems	1	1
Bowel: Distinguish temporary/permanent	1	1
Bladder problems	1	1
Bladder: Distinguish temporary/permanent	1	1
Impotence	1	1
Impotence: Distinguish temporary/permanent	1	1
Death	0	0
**Patient Experience**		
Length of treatment	1	1
Pain, fatigue, nausea	0	0
Patient testimonial	0	1
**Presentation of risk**		
Pros and Cons	1	1

Pros and Cons Quantitative	1	1
Framing	0	1
Hormone Therapy		
**Procedure**		
Description of procedure	1	1
Mentions surgical castration	1	0
Mentions estrogen	1	0
Mentions LHRH	1	0
Mentions antiandrogens	0	0
**Side Effects**		
Impotence	1	0
Impotence: Distinguish temporary/permanent	0	0
Estrogen effects	1	0
Estrogen effects: Distinguish temporary/permanent	0	0
Death	0	0
**Patient Experience**		
Palliative not curative treatment	1	0
Patient testimonial	0	0
**Presentation of risk**		
Pros and Cons	0	0
Pros and Cons Quantitative	0	0
Framing	0	0
Form/structure of presentation		
**Statistics**		
Quantitative information in text: Death	0	1
Quantitative information in text: Side effects	1	1
**Cultural sensitivity**		
Photographic or pictorial representations	0	1
Ethnic representation	0	1
Ethnicities represented	0	1
Other		
Follow-up care	1	1
Navigation tool	1	1
Personalization/Tailoring	0	0
Knowledge test	0	0
Glossary	1	1

Shared decision making	1	1
Additional resources	1	1
Non-allopathic therapies	1	0
Non-allopathic therapies: positive and negative	1	0
Mentions neutron radiation therapy	0	0
Mentions cryotherapy	0	1
Mentions chemotherapy	1	0
Uncertainty statement	1	1

Legend: 1= present, 0=absent

Note: These data were collected as part of a larger study that compared multiple prostate cancer treatment decision aids.[12]

Appendix B: IPDASi Evaluation of Decision Aids

Domain	Item	Item score (out of 4)	
		High Literacy	Plain Language
Information	DA describes the health problem (intervention or procedure) for which the index decision is required	4	4
	DA describes decision to be considered (index decision).	3	3.5
	DA describes options available for index decision	4	3.5
	DA describes rational for decision and consequences if no actions are taken	3	3.5
	DA describes positive features (benefits or advantages of each option)	2	4
	DA describes negative features (harms, side effects or disadvantages of each option	3	4
	DA makes it possible to compare the positive and negative features of available options	1	4
	DA shows negative and positive features of options with equal detail	2	4
	Information domain score (out of 100)	***47***	***99***
Probabilities	DA provides information about outcome probabilities associated with the options (ie likely consequences of decisions)	2	3
	DA specifies the defined group (reference class) of patients for which the outcome probabilities apply	2.5	2
	DA specifies the event rates for outcome probabilities (in natural frequencies)	3	2

	DA allows user to compare outcome probabilities across options using the same denominator and time period.	2	3
	DA provides information about levels of uncertainty around event or outcome probabilities	3	2
	DA provides more than one way of viewing probabilities (e.g., words, numbers, diagrams)	1	2
	DA provides balanced information about event or outcomes probabilities to limit framing biases	1	3
	Probabilities domain score (out of 100)	***31***	***63***
Values	DA describes features of options to help patients imagine experiencing physical effects	4	4
	DA describes features of options to help patients imagine experiencing psychological effects	3	3
	DA describes features of options to help patients imagine experiencing social effects	3	3
	DA asks patients to think about positive and negative features of options matter most to them	3	3
	Values domain score (out of 100)	***70***	***76***
Guidance	DA provides a step-by-step way to make a decision	3	3
	DA includes tools like worksheets or lists of questions to use to discuss options with a practitioner	3	4
	Guidance domain score (out of 100)	***45***	***80***
Plain	DA (or associated documentation) reports	1	3

Language	readability levels (using one or more of the available scales		
	Plain Language domain score (out of 100)	***0***	***72***

Note: These scores are based on the IPDASi instrument.[14]
